# Anoikis resistance and metastasis of ovarian cancer can be overcome by CDK8/19 mediator kinase inhibition

**DOI:** 10.1172/jci.insight.192113

**Published:** 2026-01-15

**Authors:** Mehri Monavarian, Resha Rajkarnikar, Emily Faith Page, Asha Kumari, Liz Quintero Macias, Felipe Massicano, Nam Y. Lee, Sarthak Sahoo, Nadine Hempel, Mohit Kumar Jolly, Lara Ianov, Elizabeth Worthey, Abhyudai Singh, Igor B. Roninson, Eugenia V. Broude, Mengqian Chen, Karthikeyan Mythreye

**Affiliations:** 1 Division of Molecular Cellular Pathology, Department of Pathology, O’Neal Cancer Center, Heersink School of Medicine, The University of Alabama, Birmingham, Alabama, USA.; 2 UAB Biological Data Science Core, The University of Alabama at Birmingham, Birmingham, Alabama, USA.; 3 Department of Pharmacology, College of Medicine, University of Arizona, Tucson, Arizona, USA.; 4 Department of Bioengineering, Indian Institute of Science, Bangalore, India.; 5 Division of Malignant Hematology and Medical Oncology, Department of Medicine, University of Pittsburgh School of Medicine, Pittsburgh, Pennsylvania, USA.; 6 Department of Neurobiology, The University of Alabama at Birmingham, Birmingham, Alabama, USA.; 7 Department of Electrical and Computer Engineering, University of Delaware, Newark, Delaware, USA.; 8 Department of Drug Discovery and Biomedical Sciences, College of Pharmacy, University of South Carolina, Columbia, South Carolina, USA.

**Keywords:** Cell biology, Oncology, Apoptosis survival pathways, Cancer, Cell stress

## Abstract

Anoikis resistance, or evasion of cell death triggered by matrix detachment, is a hallmark of cancer cell survival and metastasis. We showed that repeated exposure to suspension stress followed by recovery under attached conditions leads to development of anoikis resistance. The acquisition of anoikis resistance was associated with enhanced invasion, chemoresistance, and immune evasion in vitro and distant metastasis in vivo. This acquired anoikis resistance was not genetic, persisting for a finite duration without detachment stress, but was sensitive to CDK8/19 mediator kinase inhibition that could also reverse anoikis resistance. Transcriptomic analysis revealed that CDK8/19 kinase inhibition induces bidirectional transcriptional changes in both sensitive and resistant cells, disrupting the balanced reprogramming required for anoikis adaptation and resistance by reversing some resistance-associated pathways and enhancing others. Both anoikis resistance and in vivo metastatic growth of ovarian cancers are sensitive to CDK8/19 inhibition, thereby providing a therapeutic opportunity to both prevent and suppress ovarian cancer metastasis.

## Abstract 2

New study reveals a promising drug target (CDK8/19) that could prevent and treat ovarian<br> cancer spread by blocking cancer cells’ survival in circulation.

## Graphical abstract

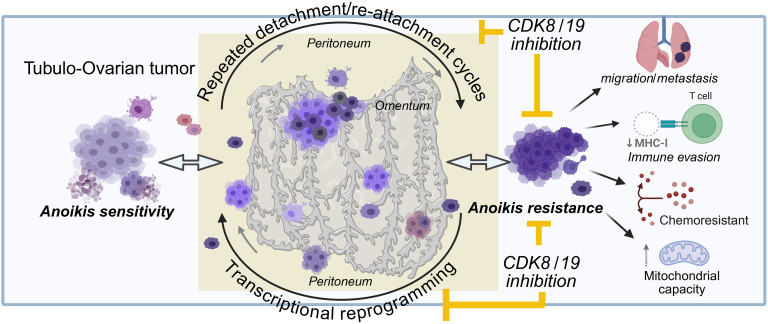

## Introduction

Metastasis causes most cancer-related deaths, yet metastatic cancers remain largely incurable. A critical step in metastasis is the ability of tumor cells to survive upon detachment from the extracellular matrix and primary tumor site, enabling their dissemination and circulation to distant sites (1–3). Despite our understanding of these metastatic features, their therapeutic targeting remains underdeveloped.

Ovarian cancers (OCs), comprising several subtypes, are among the most devastating of gynecological cancers and archetypal examples of cancers that leverage the metastatic hallmark of anoikis resistance for both transcoelomic/i.p. and distant metastasis (4–8). As malignant ascites accumulates, tumor cells must survive in suspension and evade cell death (9–12). These cells can then colonize peritoneal and mucosal surfaces or return to primary tumor sites (13). The precise mechanisms by which cells acquire such anoikis resistance remain a subject of intense investigation. Suspension culture studies have been used extensively to understand such mechanisms. However, most of them focus on single time points or long-term suspension. Despite these limitations, tumor-intrinsic signals and pathways that change the ability of cells to undergo cell death upon matrix detachment (14–18) have been identified. These anoikis resistance mechanisms include but are not limited to transcriptional upregulation of critical survival genes, repression of pro-apoptotic genes, and transient expression changes in genes associated with antioxidant defense. Changes in the genes and pathways have also been associated with tumor growth and progression in ovarian and other cancers (19–23). Coordinated regulation of specific reprogramming processes such as epithelial-mesenchymal transition (EMT) (24, 25), or cadherin/integrin switching (26), by oncogenic pathways such as Ras/Erk, PI3K/AKT, Rho, MYC, and TGF-β pathways also impacts i.p. OC survival and growth under anchorage independence (27). However, only a few studies (28, 29) directly compare anoikis-sensitive and -resistant models to understand how anoikis resistance can be reached or prevented and the mechanisms underlying this process.

Given the importance of transcriptional changes to overall cancer progression, selective and potent inhibitors of transcription-associated kinases (30) have emerged and are currently being evaluated in the clinic. Among these, CDK8 and CDK19 are of particular interest as they regulate gene expression programs in response to various cellular stresses and have shown promise in preventing resistance in other contexts (31–33). CDK8 and CDK19 are closely related kinases associated with the transcriptional mediator complex that both positively and negatively regulate transcription (34–36), and their inhibition affects different events associated with transcriptional reprogramming, including EMT (24), cell differentiation (37), and gene expression changes in response to various signals and stressors (34, 35). Notably, CDK8/19 inhibitors have reached clinical trials for solid tumors and leukemias (ClinicalTrials.gov NCT03065010, NCT04021368, NCT05052255, NCT05300438), with utility specifically for gynecological cancers under examination (38).

In this study we describe a model system that tests the effects of repeated exposures to detachment stress followed by attached regrowth to mimic potential in vivo scenarios. We delineate the impact of such repeat exposures to detachment stress on the development of anoikis stress in different OC models. Our phenotypic and transcriptomic characterization of such anoikis-resistant cells reveals nongenetic, transcriptional reprogramming, concomitant with a more aggressive phenotype in vitro and in vivo. We further show that both anoikis resistance and i.p. growth of OC can be suppressed by specific inhibition of CDK8/19 mediator kinases, which can also reverse such acquired anoikis resistance. Further, transcriptomic analysis of the effects of CDK8/19 mediator kinase inhibition reveal positive and negative changes to both core and stress-associated transcriptional responses that rebalance the transcriptional response resulting from anoikis resistance. Our findings define what we believe to be a novel therapeutic strategy for counteracting anoikis resistance and metastasis by specific targeting of CDK8/19-regulated transcriptional reprogramming.

## Results

### Attachment-detachment cycles confer anoikis-sensitive OC cells with resistance to cell death in suspension.

**Figure 1:**
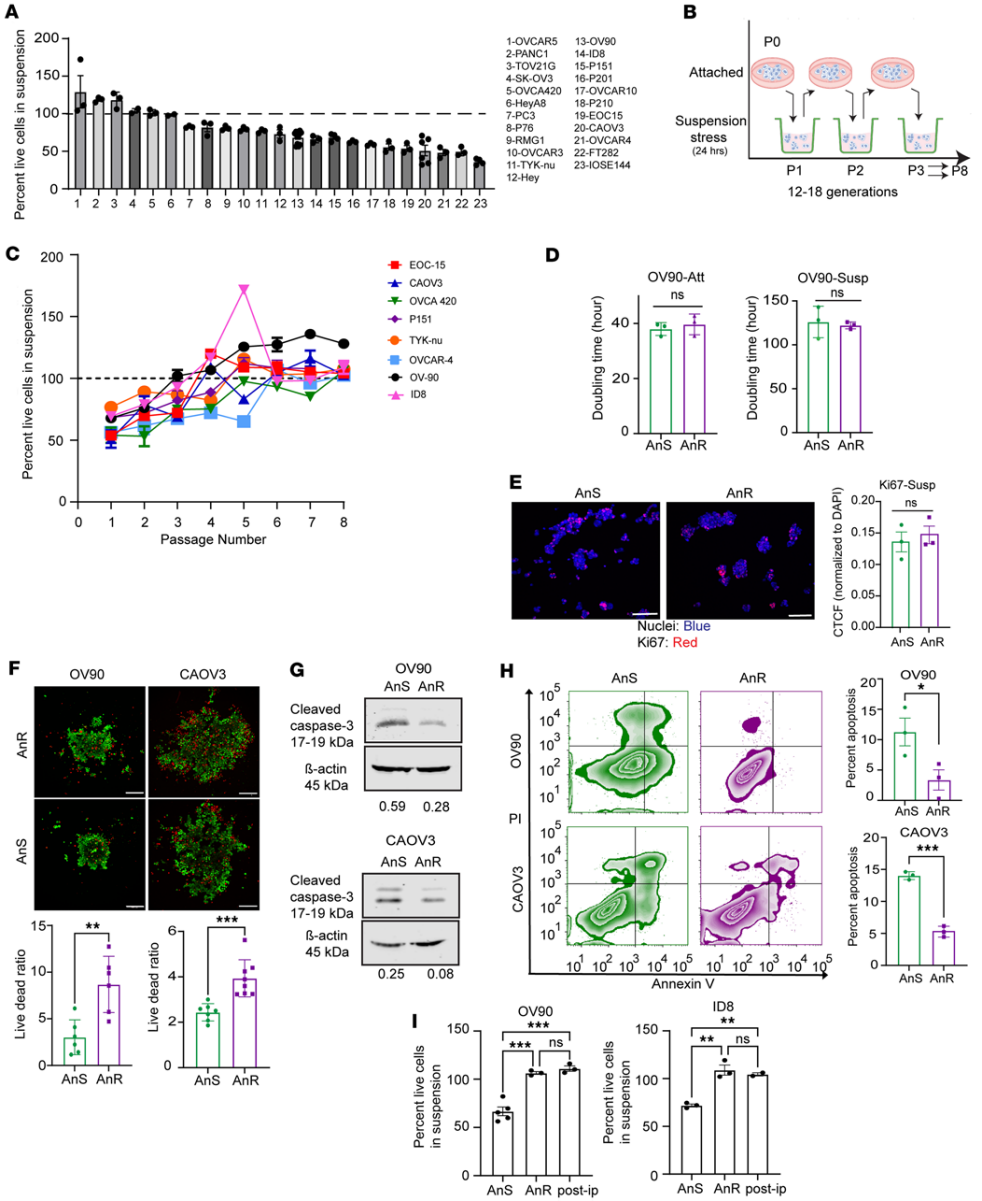
Development of anoikis resistance upon cyclic exposure to matrix detachment stress. (**A**) Percent live cells after 24 hours in suspension (trypan blue exclusion). *n* ≥ 3 per cell line. (**B**) Schematic of cyclic attached growth and suspension culture. (**C**) Percent live cells in suspension for indicated AnS cells following cyclic attachment-detachment as in **B**. (*n* = 3–12 per cell line.) (**D**) Doubling time of OV90 parental (AnS) and AnR (P7–P9) cells in attached (7 days) or suspension (10 days) culture. (**E**) Ki67 (red) normalized to DAPI in OV90 AnS and AnR cells after 24 hours in suspension. *n* = 3 with each trial including quantitation of at least 4 original magnification, 20×, fields. (**F**) Live/dead staining (Calcein AM green, live cells and ethidium homodimer; red, dead cells) of AnS and AnR cells after 24 hours in suspension (ULA plates); *n* = 6. (**G**) Representative images of cleaved caspase-3 Western blot in AnS and AnR cells after 24 hours in suspension; quantitation of cleaved caspase-3 normalized to β-actin shown below (*n* = 2). (**H**) Annexin V/PI flow cytometry (left) and quantitation (right) in AnS and AnR cells after 24 hours in suspension; *n* = 3. (**I**) Percent live cells in suspension of in vitro–derived (AnS/AnR) and mouse ascites-derived (at endpoint) OV90 and ID8 cells; *n* = 2–5. Scale bars: 100 μm (**E**), 200 μm (**F**). Data are mean ± SEM. *P < 0.05; **P < 0.01; ***P < 0.001 by 2-tailed unpaired *t* test (**D** and **E**) or 1-way ANOVA with Tukey’s test (**F**, **H**, and **I**).

To study how cells develop anoikis resistance, we first screened a panel of tumor cell lines that span commonly used OC cell line models, a pancreatic cancer cell line (PANC1), a prostate cancer cell line (PC3), primary (nonimmortalized) tumor cells from OC patient ascites (EOC15), and 3 nononcogenic immortalized ovarian surface and fallopian tube epithelial cell lines (IOSE144, FT282, and P201 and P210) (20, 23, 39). Anoikis was measured as percentage live cells in suspension relative to the initial plating numbers after plating in poly-HEMA–coated, ultra-low-attachment (ULA) conditions for 24 hours. All lines were plated at identical density in their growth media. Viability ranged from 36.1% for IOSE144 to 125.2% for OVCAR5 (Figure 1A). Models with <100% viability at 24 hours were designated anoikis sensitive (AnS); those with ≥100% were designated intrinsically anoikis resistant (AnR). A subset of AnR lines assessed for up to 72 hours retained resistance (HEYA8, PANC1, TOV21G), whereas OVCA420 increased cell death by 72 hours (Supplemental Figure 1A; supplemental material available online with this article; https://doi.org/10.1172/jci.insight.192113DS1); hence, 72 hours was used for OVCA420 in subsequent experiments. EOC15 maintained in attachment (Supplemental Figure 1B) also exhibited measurable cell death in suspension at 24 hours (Figure 1A).

We next asked whether AnS cell lines could develop resistance to loss of attachment. To simulate i.p. and distant metastasis where cells undergo detachment stress followed by attached growth (13, 40), we exposed cells to repeated attachment-detachment cycles (Figure 1B). Of AnS lines with <100% viability at first suspension exposure (P1), 7/9 human lines and 1 mouse line showed substantially reduced cell death after sequential cycles (Figure 1C), maintaining acquired AnR through additional rounds. Two lines (HEY, OVCAR3) appeared to become AnR after 3–4 cycles (Supplemental Figure 1C, HEY) but reverted to sensitivity through subsequent cycles.

We assessed if AnR was due to changes in proliferation rate or population-doubling times. No significant differences in doubling time between parental AnS and isogenic AnR cells were observed in either 2D attached or suspension conditions over 10 days (Figure 1D). Parental AnS CAOV3 could not be assessed in suspension because of extensive cell death, but no differences were seen for AnS/AnR pairs in attached growth (Supplemental Figure 1D). Ki67 staining (Figure 1E) also revealed no significant differences between the AnS and AnR cells. In contrast, live/dead staining of OV90 and CAOV3 parental AnS and isogenic AnR cells in suspension (Figure 1F) and cleaved caspase-3 (CC3) analysis after 24 hours in suspension showed reduced cell death in AnR cells as compared with AnS cells (Figure 1G). Flow cytometry for annexin V/PI indicated significantly higher live/dead ratios (2.8-fold for OV90; 1.6-fold for CAOV3) and reduced apoptosis (3.36-fold lower in OV90; 2.59-fold lower in CAOV3) in AnR versus AnS cells (Figure 1H).

To evaluate if in vitro–developed AnR mimicked in vivo AnR, we injected human OV90 cells and mouse ID8 cells i.p. into immunocompromised and immunocompetent mice, respectively. Ascites-derived cells at endpoint, expanded for 1 passage in vitro, showed resistance to suspension comparable to in vitro–adapted AnR cells (Figure 1I). These data suggest that AnR cells adapted to anoikis in vitro show an AnR phenotype similar to in vivo–adapted AnR cells from ascites.

### Acquired resistance to cell death in suspension is adaptive and reversible.

**Figure 2:**
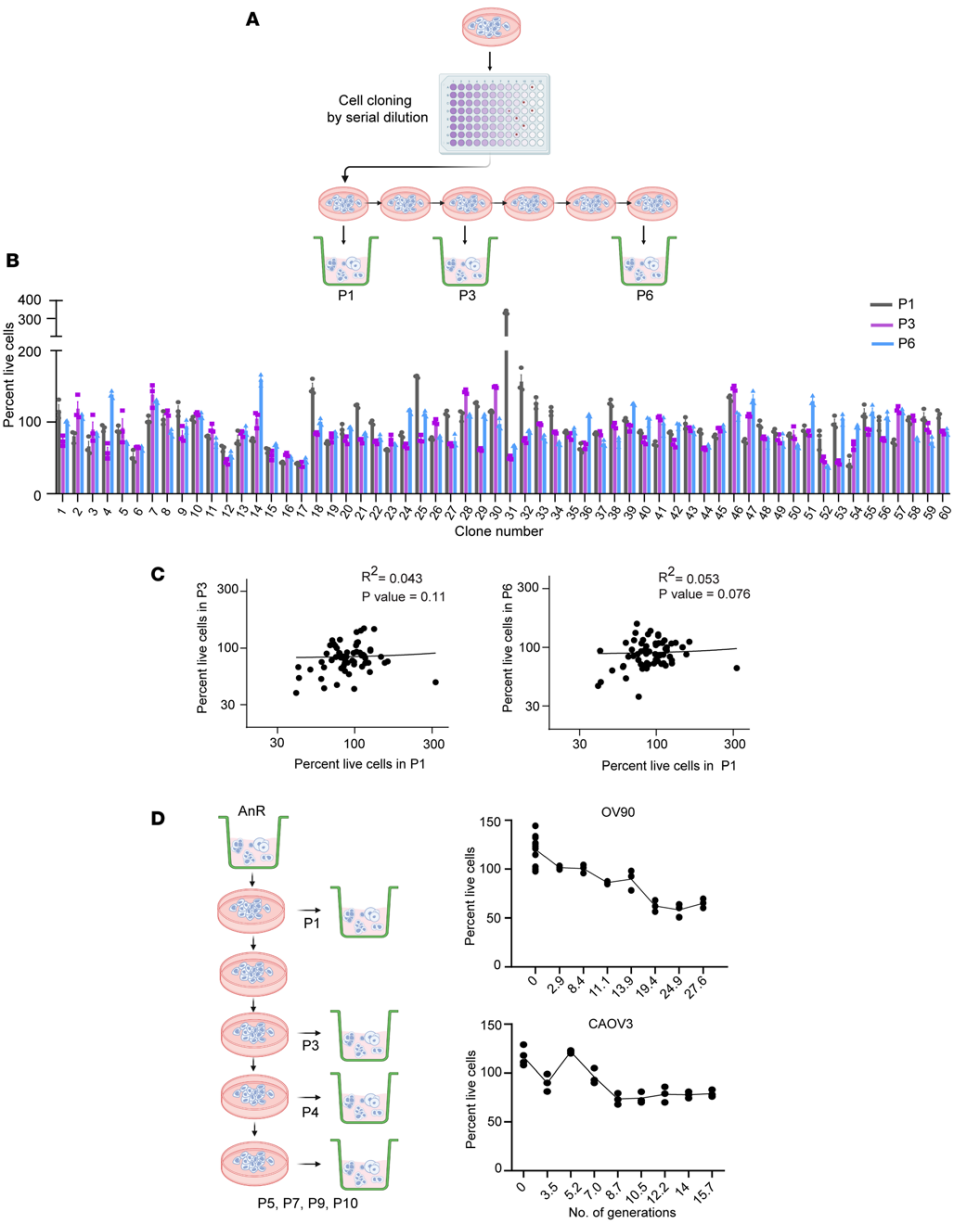
Acquired anoikis resistance represents a transient memory state in the population. (**A**) Scheme of single clone expansion (OV90) and survival assessment after 24 hours in suspension at indicated passages. (**B**) Percent survival of individual clones as in **A**; *n* = 60 clones. (**C**) Linear regression of individual clonal survival in suspension at P1 versus P3 (left) or P6 (right). (**D**) Schematic (left) and percent survival in suspension of AnR OV90 and CAOV3 cells plotted against generations in attached culture (right).

To determine whether acquired AnR in vitro was due to clonal selection, genetic mutations, or nongenetic mechanisms, we used a modified Luria-Delbrück fluctuation analysis. Recent studies have adapted the classical Luria-Delbrück test to investigate cancer drug resistance by exposing single-cell–derived clones to targeted therapy and analyzing fluctuations in surviving cell numbers to assess reversible switching between drug-sensitive and drug-tolerant states (41–46). We asked if transient switching between cellular states could similarly drive AnR. We assessed survival fluctuations in suspension across single clones from the parental/AnS OV90 population (Figure 2A; *n* = 60 single clones). Individual clones were expanded for 20 generations before determining live cell viability in suspension induced by detachment stress in poly-HEMA–coated, ULA plates (Figure 2, A and B; P1 survival, gray bars). Clones were also maintained in 2D cultures, and viability of the expanded clonal populations was measured after 3 (P3) and 6 (P6) passages in 2D (Figure 2, A and B). If clones switched between sensitivity (<100% survival) and resistance (≥100%), it would indicate nonheritable resistance. We observed no significant correlation in survival over time among clones (Figure 2C), which had doubling times ranging from 39 to 62 hours (Supplemental Figure 2A; *n* = 10 random clones), indicating the absence of fixed clonal states. Mean survival fractions (~0.9) and interclonal fluctuations as quantified by the coefficient of variation (CV: 0.25–0.3) were consistent across different passages (CV values for P1 = 0.283 ± 0.05, P3 = 0.289 ± 0.05, and P6 = 0.259 ± 0.05), with observed fluctuations exceeding those of the parental population (CV = 0.11 ± 0.04, obtained from *n* = 11 biological repeats), where ± denotes the 95% confidence interval of the CV obtained from bootstrapping. If cells responded purely randomly to stress, fluctuations across clones would mirror population noise. However, the higher CV in clones suggests a memory effect or the presence of prestress states influencing detachment stress responses and anoikis resistance.

To test this memory effect, we evaluated the stability of the AnR state in OV90 and CAOV3 cells by propagating AnR cells in attached growth for several generations without suspension stress. When rechallenged with suspension stress, OV90 cells regained anoikis sensitivity after 11–14 generations and CAOV3 cells after 8–9 generations, approximating parental population sensitivity levels (Figure 2D). We applied previously developed analytical formulas (43, 45, 46) to predict what levels of fluctuations would be expected from switching between an AnS state (cell death in suspension) and an AnR state (cell survival and proliferation in suspension). If *f* is the fraction of cells in the resistant state, *fe*^γT^ = 0.9, where *T* = 24 *hr* and growth rate 


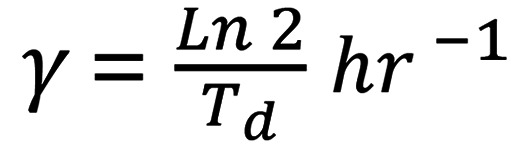


where *T_d_* is the cell-doubling time of AnR cells in suspension that is experimentally determined. Thus, given a value of *T_d_*, the fraction of resistant cells can be computed from the above equation, and hence a suspension doubling time of 100 hours for OV90 in suspension (Figure 1D) results in *f* ≈ 0.76. We used equations derived previously to obtain predicted fluctuations (46). Given clonal expansion before the first survival test in suspension and a transient heritability of 10–11 generations for the resistant state (Figure 2D), the model-predicted fluctuations were much less than 0.01 and 30-fold lower than the observed values (0.25–3) for all biologically relevant values of *T_d_* (≥38 hours, OV90 2D doubling time; Figure 1D). Thus, reversible switching between these 2 phenotypic states cannot explain the observed clone-to-clone variations in surviving cells.

To uncover mechanisms underlying interclonal fluctuations, we calculated the effective growth rate during the first suspension test using *Ln*(N_t_/N_0_)/24, where N_0_ and N_t_ are cell numbers at the start and end of the 24-hour suspension period. Clones exhibited significant variation in growth rates, averaging –0.004 hr^–1^ and a CV of 300%. Approximately two-thirds of clones showed negative growth rates (N_t_ < N_0_), while one-third had positive growth rates (N_t_ > N_0_), with 4 clones doubling in suspension (doubling time: 45 hours), similar to measurements in 2D cultures. These high clonal growth rate variations in suspension could not be captured by a simple 2-state model. Together with the lack of concordance between the predicted and observed clonal fluctuation, these data suggest a continuum of cellular states but are, however, consistent with nongenetic effects on adaptation of cells as they develop resistance to the biological stress of loss of attachment (anoikis resistance). Nongenetic adaptation was confirmed by whole-exome sequencing. The total number of genes that were mutated in any given replicate was found to be 2,860. However, no significant differentially mutated genes (using a *P* < 0.05 and Fisher’s exact test) were found between the 2 isogenic populations of cells (top 50 mutated genes based on total number of mutations present in OV90 cells are shown in Supplemental Figure 2B). Thus, acquired resistance was not due to clonal selection of an AnR subpopulation that preexisted, but rather represents the development of an anoikis-tolerant state.

### Adapted anoikis-tolerant OC cells are more chemoresistant and metastatic in vivo.

**Figure 3:**
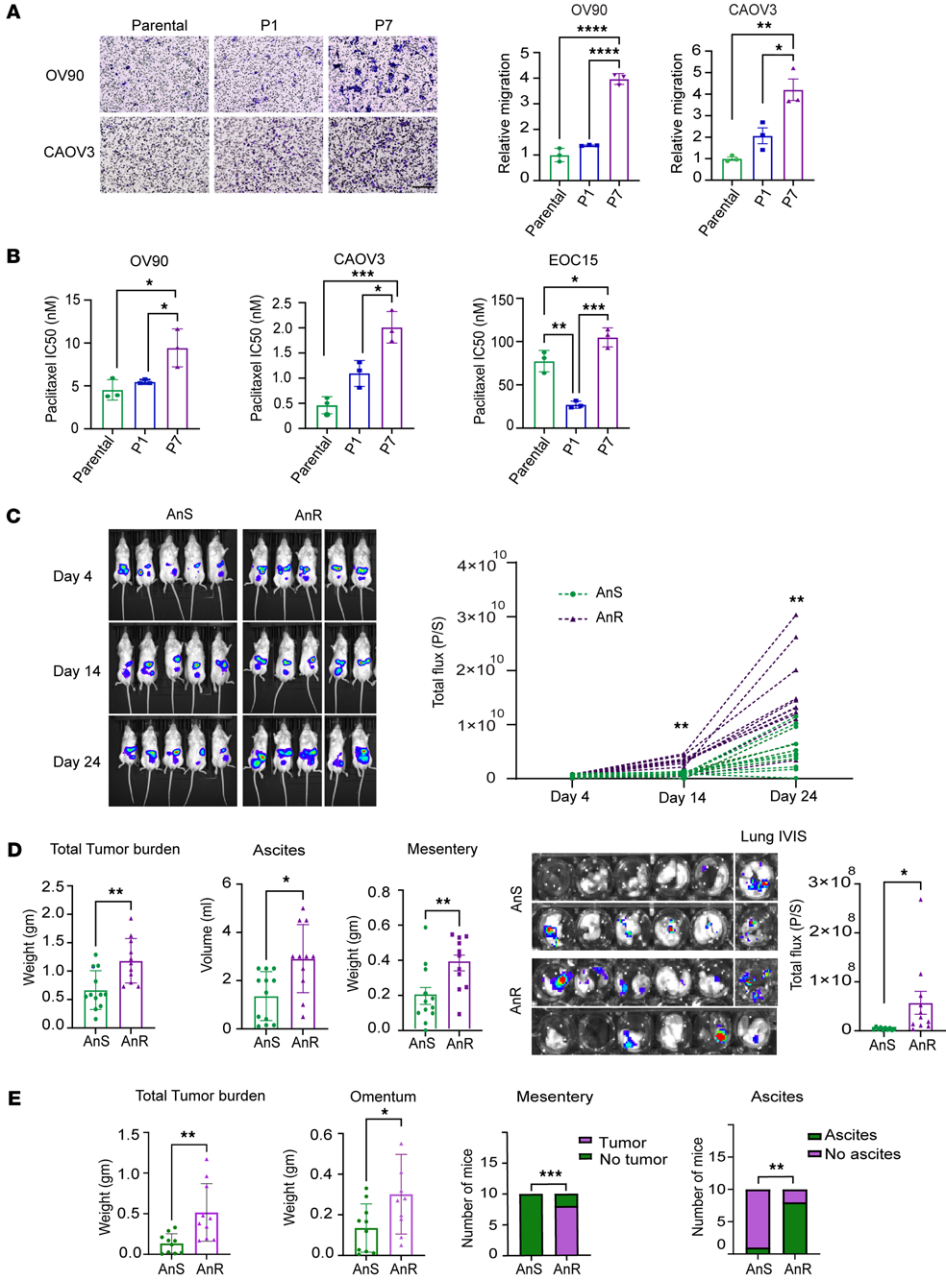
Acquired anoikis resistance leads to increased in vitro migration and chemoresistance and in vivo i.p. growth and metastasis. (**A**) Transwell migration on fibronectin of parental, P1, and P7 (AnR) cells after 24 hours; *n* = 3. (**B**) IC_50_ to paclitaxel in OV90, CAOV3, and EOC15 cells after 72 hours (Sulforhodamine B [SRB; Thermo Fisher Scientific, catalog A14769.06] assay); *n* = 3. (**C**) Whole body bioluminescence (BLI) of NOD/SCID mice injected i.p. with 5 × 10^6^ OV90 parental (P0) or AnR (P7) cells. (**D**) Tumor weight, ascites volume, mesenteric weight, and lung BLI from mice receiving OV90 P0 (AnS) or P7 (AnR) cells at day 39–40; *n* = 11–12. (**E**) Tumor weight, omental weight, and no. of mice with tumors in the mesentery and with retrievable ascites from C57BL/6J mice injected i.p. with parental or AnR ID8 cells at day 80–82; *n* = 10. *P < 0.05; **P < 0.01; ***P < 0.001; ****P < 0.0001 by 1-way ANOVA with Tukey’s test (**A** and **B**), 2-way ANOVA with Tukey’s test (**C**), or 2-tailed unpaired *t* test (**D** and **E**). Data are mean ± SEM.

Prior studies have implicated anoikis resistance as a phenotype of metastatic and chemoresistant cells (5, 47–49). Given that reversion to the original AnS state occurred in the absence of stress (Figure 2D), we tested if such an acquired “tolerance” was sufficient to alter properties associated with tumorigenicity. Transwell migration assays revealed that OV90-AnR cells (P7) underwent significantly higher migration through fibronectin compared with the parental population (P0) and cells after single suspension exposure (P1) (Figure 3A). Increased migration was also observed in CAOV3-AnR cells (Figure 3A) and additional isogenic pairs including EOC15, p151, and OVCA420 (Supplemental Figure 3A), demonstrating increased motility as a feature of acquired AnR. Chemosensitivity testing revealed that AnR cells across models had significantly higher IC_50_ concentrations for paclitaxel (Figure 3B), including in suspension cultures (Supplemental Figure 3B), though no significant differences were observed for doxorubicin or cisplatin (Supplemental Figure 3C) in such an acute response test.

To assess if acquired AnR increases i.p. growth and metastasis of tumor cells, we injected luciferase-expressing OV90 parental cells (P0), or cells expanded under attached conditions after development of AnR (P7), into the peritoneal cavity of NOD/SCID mice. Bioluminescence imaging (BLI) revealed significantly increased tumor burden over time in mice receiving AnR cells as compared with mice receiving parental (P0) cells (Figure 3C). BLI data are presented until day 24, as ascites accumulation beginning ~day 25 precluded subsequent reliable imaging. The study was terminated at day 39–40, when P7/AnR recipient mice became moribund. Endpoint i.p. tumor weight in AnR recipient mice was twice that of parental-recipient mice, with higher ascites volume and mesenteric tumor burden (Figure 3D). Strikingly, AnR recipient animals showed higher lung metastatic growth by BLI of explanted lung tissues (Figure 3D).

Since the in vitro–derived AnR cells mirrored suspension survival of in vivo–derived AnR cells of human (OV90) and mouse (ID8) origin (Figure 1I), we tested if acquired anoikis resistance was sufficient to increase i.p. growth and metastasis of tumor cells even in the presence of the immune system. We injected ID8 parental cells (parental/P0) or in vitro–adapted AnR cells (AnR/P7) into the peritoneal cavity of C57BL/6J mice. The study was terminated when mice from the P7/AnR ID8 group exhibited signs of being moribund. Adapted ID8 AnR cells produced significantly higher disease burden as evident from the higher overall measurable tumor burden (Figure 3E), including in the omentum and mesenteric regions and from the number of mice with measurable ascites (Figure 3E). Lung metastasis, however, was not evident from pathological assessments in the ID8 model. These data demonstrate that repeated cycles of exposure to suspension stress followed by attached growth lead to adaptation that is sufficient to promote aggressive disease in vivo mimicking disease spread seen in patients with advanced-stage OC.

### Development of adaptive anoikis resistance is concomitant with transcriptional reprogramming over time.

**Figure 4:**
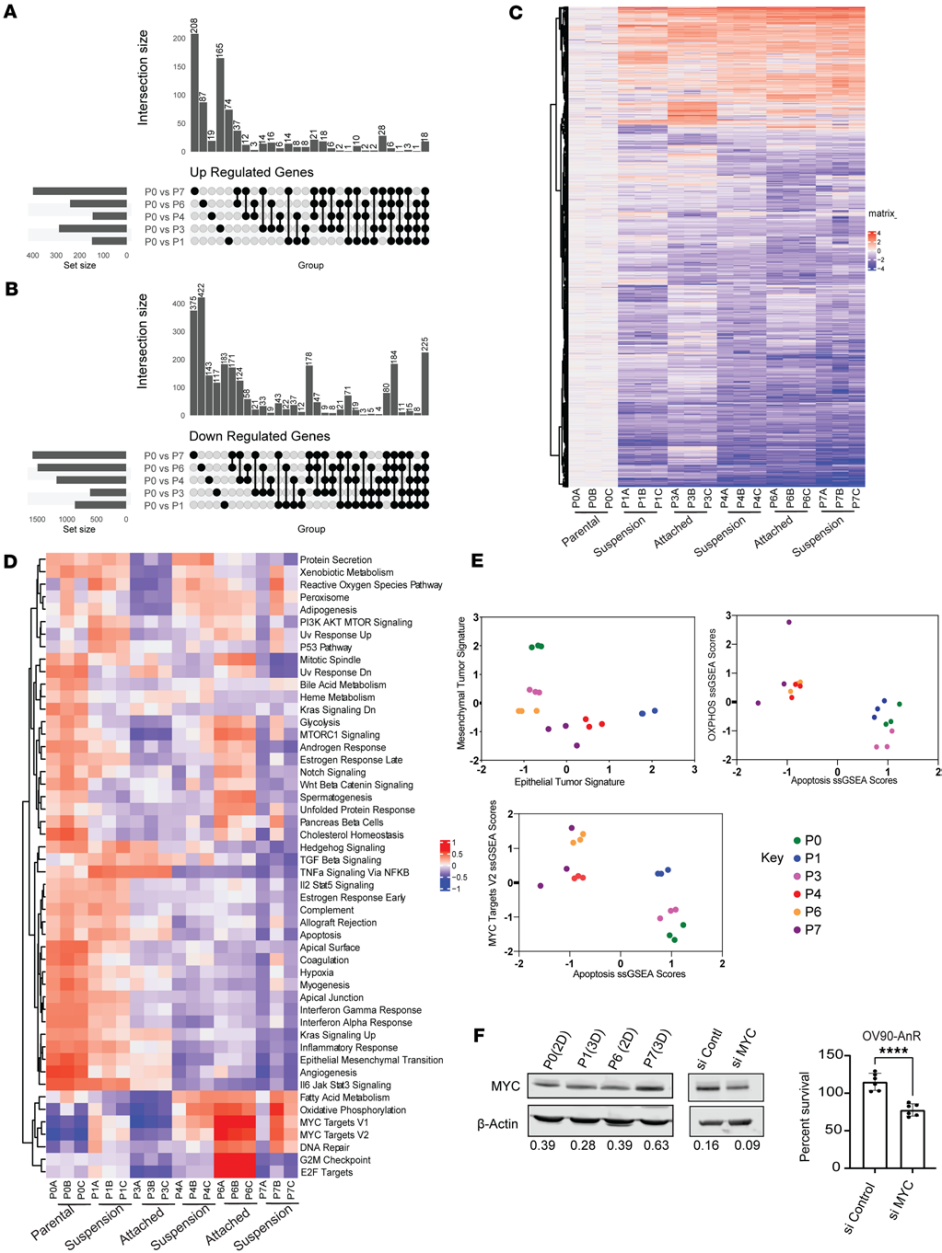
Transcriptional changes and pathway alterations during adaptation to anoikis over time. (**A** and **B**) UpSet plots of upregulated (**A**; *P* < 0.05, L2FC > 1.5) and downregulated (**B**; *P* < 0.05, L2FC < –1.5) genes in OV90 across P0 vs. P1, P3, P4, P6, and P7 comparisons. (**C**) Heatmap of DEGs (L2FC) for individual replicates (P0–P7) clustered by Euclidean distance; attached and suspension time points indicated. (**D**) GSVA heatmap of hallmark pathway normalized enrichment scores (NES) for OV90 samples (P0–P7) clustered by Euclidean distance. (**E**) Scatterplots of NES for epithelial-mesenchymal (left), apoptosis vs. oxidative phosphorylation (right), and apoptosis vs. MYC targets V2 (bottom) across passages. ssGSEA, single-sample gene set enrichment analysis. (**F**) Western blot for c-MYC in indicated OV90 cells (left), c-MYC knockdown validation (middle), and percent survival in suspension of P7 OV90 cells after siMYC knockdown (right). ****P < 0.0001, 2-tailed unpaired *t* test. Data are mean ± SEM.

We next assessed the transcriptional changes in response to suspension stress and during recovery periods of attached growth. Bulk RNA sequencing of OV90 and CAOV3 cells undergoing adaptation at various suspension and recovery time points revealed that the number of differentially expressed genes (DEGs; defined by padj <= 0.05, L2FC > 1.5) after the first exposure to suspension stress (P0 versus P1 after 24 hours in suspension) was 1,011 for OV90 and 362 for CAOV3, of which a total of 148 were upregulated and 863 downregulated for OV90 and 171 and 191 for CAOV3, respectively (Figure 4, A–C, and Supplemental Figure 4, A–C). In the case of OV90, 74 of the 148 upregulated genes were upregulated only during the first exposure to stress (P0–P1) (Figure 4A). As cells adapted to stress in subsequent cycles, the total number of DEGs decreased over time, with more genes downregulated than upregulated in both OV90 and CAOV3 cell lines (Figure 4, A and B, and Supplemental Figure 4, A and B). The highest number of unique upregulated genes was observed from P0 to P7 (208 genes, OV90) and during the transition to resistance in OV90 cells (P0 and P3 time points; 165 genes). Most of the unique downregulated genes were found between P0 versus P7 (suspension AnR) and P0 versus P6 (attached AnR), with 375 and 422 genes, respectively. Of these genes, 225 were consistently downregulated over time (Figure 4B, last column). For CAOV3, the comparison between P0 and P6 (2D comparisons of AnS and AnR) had a total of 131 upregulated genes and 213 downregulated genes, and 29 upregulated and 115 downregulated were unique to this comparison (Supplemental Figure 4, B and C). The P0 versus P7 comparison gave the greatest number of uniquely up- and downregulated genes (110 and 379, respectively) in CAOV3 (Supplemental Figure 4, A–C), similarly to OV90. Thus, while the number of DEGs per time point varied between the cell lines (Figure 4, A–C, and Supplemental Figure 4, A–C), the number of differentially downregulated genes exceeded the number of differentially upregulated genes in both models. The magnitude and timing of changes also differed between cell lines, suggesting cell line–specific adaptation kinetics. Together these data demonstrate the extent of transcriptional changes occurring after the first exposure to stress, with only a subset of the same changes maintained in later stages of adaptation.

Gene set variant analysis (GSVA) of the different time points using the Human Molecular Signatures Database (MSigDB) Hallmark Collections revealed a subset of hallmarks in OV90 cells that were positively enriched during the adaptation cycles, such as those associated with TGF-β signaling, Hedgehog signaling, and TNFa signaling via NFkb (Figure 4D). Later passages showed reduced apoptosis (Figure 4, D and E), and the cells alternated between epithelial and mesenchymal states before converging toward a hybrid EM state once adapted (Figure 4E). Additional pathways were more specific to attached growth conditions in the adapted resistant stages in OV90 cells, such as mTORC1, Notch, and Wnt beta catenin (Figure 4D, P6). GSVA in CAOV3, which was more anoikis sensitive than OV90 (Figure 1), had a different mutational background than OV90 (https://depmap.org/portal/), and reverted back to sensitivity in fewer passages as compared with adapted OV90 AnR cells (Figure 2D), identified overlapping and some distinct enrichments as compared with OV90. Hallmarks including TGF-β signaling, hypoxia, Hedgehog signaling, and TNFa signaling via NFkb that were enriched during adaptation in OV90 cells remained enriched in CAOV3 even at later time points (Supplemental Figure 4D), suggesting differences in the timing and maintenance of the pathway activation between the 2 models. Both cell lines, however, showed common enrichments in oxidative phosphorylation and MYC targets in adapted cells (Figure 4E and Supplemental Figure 4D). Increase in *MYC* RNA levels (Supplemental Figure 4E) was also manifested at the protein level (Figure 4F), with lowering MYC in the adapted AnR OV90 cells using siRNA leading to reduced survival in suspension (Figure 4F). Together, these data highlight common mechanisms enriched in adapted cells, with additional pathways altered at different times between models, during adaptation.

### Adapted AnR cells depend on oxidative phosphorylation for survival.

**Figure 5:**
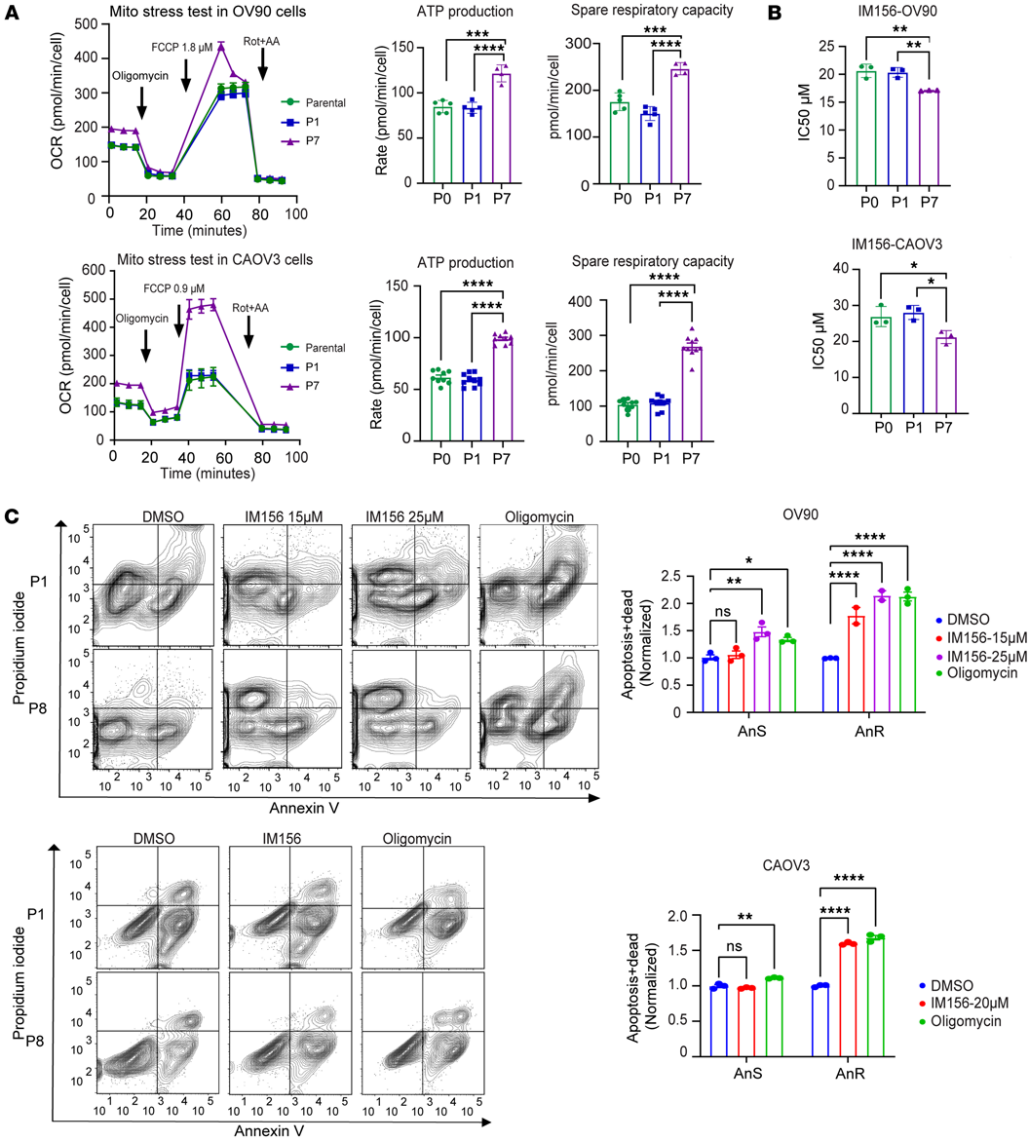
Adapted AnR cells are dependent on OXPHOS for enhanced survival upon loss of attachment. (**A**) Oxygen consumption rate (OCR), ATP production, and spare respiratory capacity (SRC) in OV90 and CAOV3 P0, P1, and P7 cells measured by Seahorse XF96 Mito Stress Test (oligomycin 1.5 μM, carbonyl cyanide *p*-trifluoromethoxyphenylhydrazone [FCCP] 1.8 μM for OV90/0.9 μM for CAOV3, rotenone/antimycin A 0.5 μM); (**B**) IC_50_ to IM156 in OV90 and CAOV3 cells from indicated passages after 96 hours (SRB assay); *n* = 3. (**C**) Annexin V/PI flow cytometry of OV90 (top) and CAOV3 (bottom) cells from indicated passages after 72 hours’ treatment with DMSO, IM156, or oligomycin in suspension; *n* = 3. *P < 0.05; **P < 0.01; ***P < 0.001; ****P < 0.0001 by 1-way ANOVA with Tukey’s test (**A** and **B**) or 2-way ANOVA with Tukey’s test (**C**). Data are mean ± SEM.

Changes in the oxidative phosphorylation (OXPHOS) pathway were a hallmark in both OV90 and CAOV3 adapted AnR cells (Figure 4, D and E, and Supplemental Figure 4D). We therefore tested for direct effects on mitochondrial respiration using extracellular flux analysis (Seahorse XF96) (Figure 5A). Comparing parental cells (P0) with cells after 1 round of suspension stress (P1) or adapted AnR/P7 cells, we found that AnR cells from both lines exhibited higher baseline oxygen consumption and extracellular acidification rates (Supplemental Figure 5). ATP-dependent OCR, measured by adding oligomycin to inhibit complex V (ATP synthase), was significantly higher in P7/AnR cells compared with P0 and P1 in both OV90 (mean: P0 = 84.8, P1 = 83.4, P7 = 121.5 pmol/min/cell) and CAOV3 (mean: P0 = 61.8, P1 = 59.2, P7 = 98.9 pmol/min/cell) (Figure 5A). SRC, representing mitochondrial ability to produce energy beyond basal maintenance and shown to increase during malignant transformation and invasion (50), was determined by adding FCCP to uncouple OXPHOS and drive OCR to maximal levels. SRC was significantly higher in AnR/P7 cells compared with P0 and P1 in both models (Figure 5A).

Since adapted AnR cells showed higher SRC, we compared the sensitivity of parental (P0) and P1 cells and AnR /P7 cells with the biguanide IM156, an AMPK activator and complex I inhibitor currently in clinical trials (51). Remarkably, we found that adapted AnR cells from P7 for both OV90 and CAOV3 cells showed significantly lower IC_50_ to IM156 compared with parental and P1 cells (OV90 mean IC_50_: parental= 20.6, P1= 20.3, P7 = 17.1 μM, and CAOV3 mean IC_50_: parental= 26.9, P1= 28, P7= 21.2 μM; Figure 5B). Moreover, a sub-IC_50_ dose of IM156 induced cell death in suspension (anoikis) to a significantly greater extent in the adapted AnR/P7 cells as compared with the parental counterpart (Figure 5C). Similarly, inhibition of the ETC complex V using oligomycin was able to resensitize and induce anoikis to a significantly higher degree in the adapted AnR/P7 cells as compared with parental cells (Figure 5C). These data demonstrate that adapted AnR cells have developed an enhanced mitochondrial capacity to support their superior survival in suspension and indicate that blocking OXPHOS in these cells could reverse such an adaptive phenotype.

### Adapted AnR cells evade immune surveillance.

**Figure 6:**
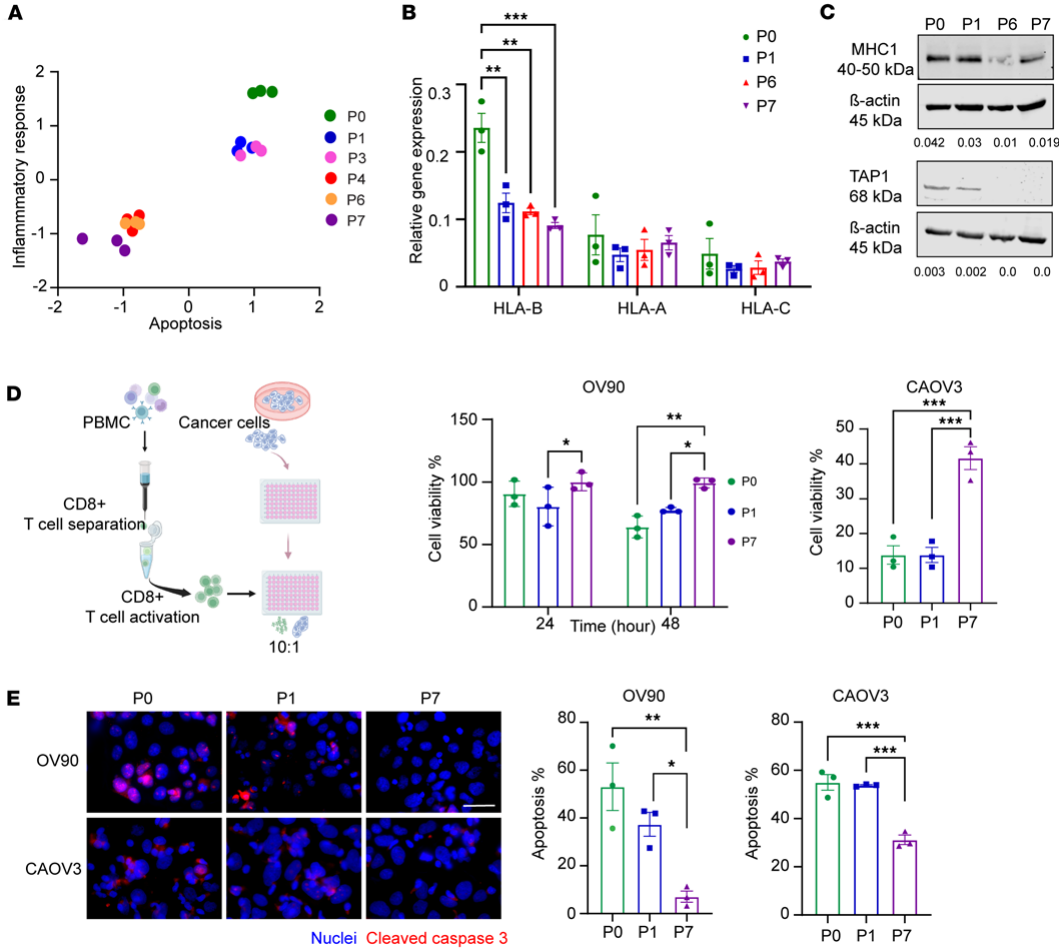
Adapted AnR cancer cells evade killing by cytotoxic T cells. (**A**) NES for inflammatory response vs. apoptosis hallmarks in OV90 cells across passages P0–P7; *n* = 3. (**B**) qRT-PCR of *HLAA*, *B*, and *C* in AnS (P0, P1) and AnR (P6, P7) OV90 cells; *n* = 3. (**C**) Representative Western blot for TAP1 and MHCI in AnS and AnR cells; quantitation normalized to β-actin below (*n* = 2). (**D**) Schematic (left) and percent viability (right) of OV90 and CAOV3 cells after coculture with activated human CD8^+^ T cells (24–48 hours for OV90, 24 hours for CAOV3); *n* = 3. (**E**) Cleaved caspase-3 immunofluorescence in OV90 and CAOV3 cells after CD8^+^ T cell coculture (48 hours for OV90, 12 hours for CAOV3); scale bar: 50 μm; *n* = 3. *P < 0.05; **P < 0.01; ***P < 0.001; by 2-way ANOVA with Tukey’s test (**B**, **D**-OV90), 1-way ANOVA with Tukey’s test (**D**-CAOV3, **E**). Data are mean ± SEM.

Bulk RNA-seq results also revealed perturbation in pathways that may impact immune recognition of the adapted AnR cells compared with their sensitive counterpart. Of note, the inflammatory response pathway was enriched early during adaptation (P0 [parental], P1 and P3 [collected after 1 and 3 cycles of detachment, respectively]) in comparison with later stages of adaptation (P4, P6, and P7 [collected after 4, 6, and 7 cycles of detachment, respectively]) in OV90 cells (Figure 6A). We also examined expression of HLA genes, as downregulation of major histocompatibility complex I (MHCI) antigen presentation is a known mechanism of immune evasion in multiple cancers (52) and has been associated with anoikis resistance in other cancers (49). We found that *HLAB* as part of MHCI, but not *HLAA* and -*C*, was downregulated over time during development of adaptive AnR in the attachment-detachment cycles in OV90 cells (Figure 6B). Protein levels of MHCI protein were also lower in adapted P6 and P7 cells compared with P0 and P1 cells (Figure 6C). Additionally, TAP1 (transporter associated with antigen processing 1), which is involved in MHCI antigen presentation, was absent in adapted AnR cells (Figure 6C). TAP1 and MHCI play crucial roles in tumor antigen recognition by cytotoxic CD8^+^ T cells (53). We thus hypothesized that adapted AnR cells that have reduced expression of MHCI and TAP1 could be less susceptible to immune-mediated killing compared with their AnS counterparts. We isolated and activated CD8^+^ T cells from PBMCs derived from healthy individuals (Figure 6D) and added them to OV90 and CAOV3 cells from P0, P1, and P7 cells cultured in regular attached conditions (Figure 6D). We found a time-dependent decrease in cell viability of the P0 OV90 (parental population) as compared with the adapted AnR OV90 cells (Figure 6D). For CAOV3 cells, we observed a much stronger and faster T cell killing response compared with OV90s, as at 24 hours, only 10% of viable cells remained in the parental AnS cells (P0, P1). In contrast, 40% of adapted AnR cells (P7) survived (Figure 6D). To measure apoptosis of tumor cells induced by CD8^+^ T cells, we examined CC3 after 48 hours of coculture of OV90 cells with T cells and 12 hours of coculture for CAOV3 cells because of their higher sensitivity (Figure 6E). We found higher CC3 in AnS cells (P0, P1) as compared with the isogenic AnR cells (P7) (Figure 6E). These data suggest that adaptation to detachment-induced cell death in OC cells facilitates immune evasion concomitant with reduced antigen presentation, likely through the observed downregulation of MHCI and TAP1.

### Inhibiting the transcriptional mediator kinase CDK8/19 suppresses anoikis resistance in vitro and i.p. growth in vivo.

**Figure 7:**
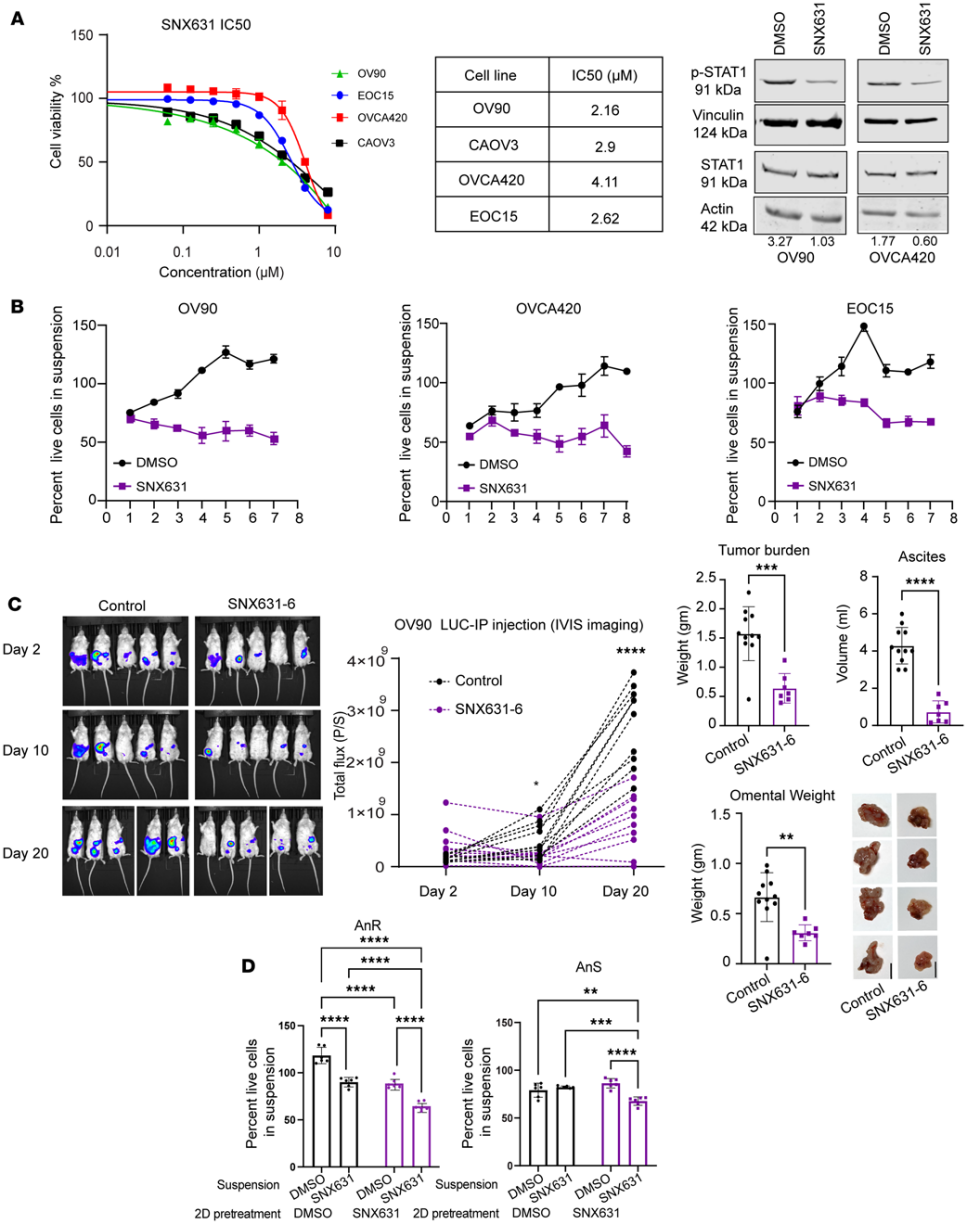
CDK8/19 mediator kinase inhibition prevents development of anoikis resistance and i.p. xenograft tumor growth. (**A**) IC_50_ of indicated cell lines after 7 days with SNX631 (SRB assay) and Western blot of pSTAT1/STAT1 normalized to their respective housekeeping controls from cells treated with 500 nM SNX631 for 24 hours. (**B**) Percent survival of AnS cells in suspension following cyclic attachment-detachment as in Figure 1B with DMSO or 500 nM SNX631; *n* = 3. (**C**) Whole body BLI (left, middle) (*n* = 11–12) and endpoint tumor weight, ascites volume, and omental weight and pictures (right) (day 41, *n* = 7–11) from NSG mice injected i.p. with 5 × 10^6^ live OV90 cells receiving control or SNX631-6–medicated diet. (**D**) Percent survival of AnR and AnS OV90 cells pretreated with DMSO or 500 nM SNX631 for 96 hours in 2D followed by 24 hours in suspension ± SNX631; *n* = 6/condition. *P < 0.05; **P < 0.01; ***P < 0.001; ****P < 0.0001 by 2-way ANOVA with Tukey’s test (**B**, **C** BLI, **D**) or 2-tailed unpaired *t* test (**C** endpoint). Data are mean ± SEM.

Transcriptional changes were both overlapping and distinct between the 2 cell line models, including changes in timing of the pathway enrichment. We thus tested the effects of inhibiting the kinase activity of CDK8/19, a pleiotropic regulator of transcriptional reprogramming, on development of adaptive AnR using SNX631 as a selective inhibitor of CDK8/19 (54). We first assessed sensitivity of intrinsically AnS cell lines (OV90, CAOV3, EOC15, and OVCA420) to SNX631 over 7 days, as CDK8/19 inhibition has been reported to be cytostatic in some cancers. IC_50_s ranged from 2.16 μM (OV90) to 4.1 μM (OVCA420, Figure 7A), over 2 orders of magnitude higher than SNX631’s IC_50_ for CDK8/19 inhibition in a cell-based assay (11 nM) (54). Hence, CDK8/19 activity did not appear to be required for proliferation of these cell lines in vitro. Given the stress associated with matrix detachment, we tested SNX631’s effects on anoikis adaptation. For this, we chose a 500 nM concentration of SNX631, which does not inhibit cell growth but is sufficient for complete CDK8/19 inhibition in a cell-based assay (55) and reducing the phosphorylation of STAT1 at S727 (Figure 7A), which is phosphorylated directly (but not exclusively) by CDK8/19 (56, 57). We found that treatment of OV90, OVCA420, and immortalized EOC15 cells with 500 nM SNX631 during cyclic attachment-detachment culture cycles prevented the development of AnR, with the mean percentage of live cells reaching a maximum of only 53.28%, 63.7%, or 67%, respectively, for each of the models, while DMSO-treated control cells reached or exceeded 100% (Figure 7B). These data demonstrate that SNX631 prevented the development of AnR. To assess whether global downregulation of overall transcription rather than transcriptional reprogramming is sufficient to block the development of anoikis resistance, we treated OV90 cells during cyclic culture of attachment-detachment with THZ1, which is a covalent CDK7 inhibitor that also targets CDK12 and was shown to globally downregulate transcription (58). Prior studies established that THZ1 potently inhibits CDK7 with an IC_50_ of ~3.2 nM in vitro, with >50% inhibition in cellular assays achieved in the range of 10–40 nM (58). We found that in contrast with CDK8/19 inhibition, a sublethal concentration of THZ1 that is known to be effective at CDK7 inhibition (58) and chosen based on IC_50_ in OV90s (Supplemental Figure 6A), had no impact on development of AnR (Supplemental Figure 6B). These data suggest selective dependence on CDK8/19 rather than overall transcription during development of AnR.

Since development of AnR in vitro mimicked development of AnR in vivo (Figure 1I), to test whether CDK8/19 inhibition also affects in vivo i.p. growth of OC cells, we injected OV90 parental/AnS luciferase-expressing cells into the peritoneal cavity of NSG mice. Mice were randomized in 2 groups, one receiving SNX631-6 (an equipotent analog of SNX631 and a clinical drug candidate, administered in medicated diet (at 350 ppm) (31, 38, 55); the second group received control diet. Whole body BLI revealed a significant decrease in overall i.p. tumor burden over time in mice receiving SNX631-6 (Figure 7C). The study was conducted until day 41, with BLI data shown until day 20 (Figure 7C), as ascites buildup in the control group starting at ~week 4 after tumor cell injection interfered with accurate luciferase detection in animals. Endpoint total tumor burden, ascites volume, and omental tumors were significantly reduced in size, volume, and weight in mice receiving SNX631-6 (Figure 7C). These results indicate that inhibition of CDK8/19-regulated transcriptional reprogramming prevented and suppressed anoikis resistance in vitro and i.p. tumor growth and metastasis in vivo.

We next wanted to test if SNX631 could also resensitize AnR cells to anoikis. Since OC cells tested were not sensitive to SNX631 in steady-state 2D growth (Figure 7A) and did not induce significant changes in cell death when SNX631 was added at the first exposure to suspension (P1 DMSO vs. SNX631, Figure 7B), we tested if pretreatment of attached cells with SNX631 followed by suspension culture for 24 hours, with or without SNX631, would affect cell death. We found that pretreatment of SNX631 in 2D reduced the survival of adapted OV90 AnR cells in suspension to the level of their AnS parent cells (Figure 7D, 2D SNX suspension DMSO condition). Pretreatment with SNX631 (2D) followed by maintaining the treatment in suspension culture further increased the cell death of adapted OV90 AnR cells in suspension by as much as 40% (Figure 7D, 2D SNX suspension SNX condition). In contrast, pretreatment (2D) of parental OV90 AnS cells with SNX631 did not significantly increase cell death in suspension (Figure 7D, 2D SNX suspension DMSO condition). However, when SNX631 was also present during suspension culture, AnS cells exhibited 25% more cell death (Figure 7D, 2D SNX suspension SNX condition). Strikingly, vehicle-pretreated AnR cell (2D) with SNX631 added only in suspension exhibited significantly higher cell death (Figure 7D, 2D DMSO suspension SNX condition) as well, as compared with parental AnS cells under the same conditions, which exhibited no significant change in cell death (Figure 7D, 2D DMSO suspension SNX condition, and seen in Figure 7B, P1). These data together suggest that SNX631 promotes anoikis sensitivity with a heightened vulnerability of AnR cells to such CDK8/19 inhibition and demonstrates that CDK8/19 inhibition can both prevent and reverse AnR.

### CDK8/19 inhibition disrupts the balanced transcriptional reprogramming underlying both the development of anoikis resistance and the acute response to anoikis stress.

**Figure 8:**
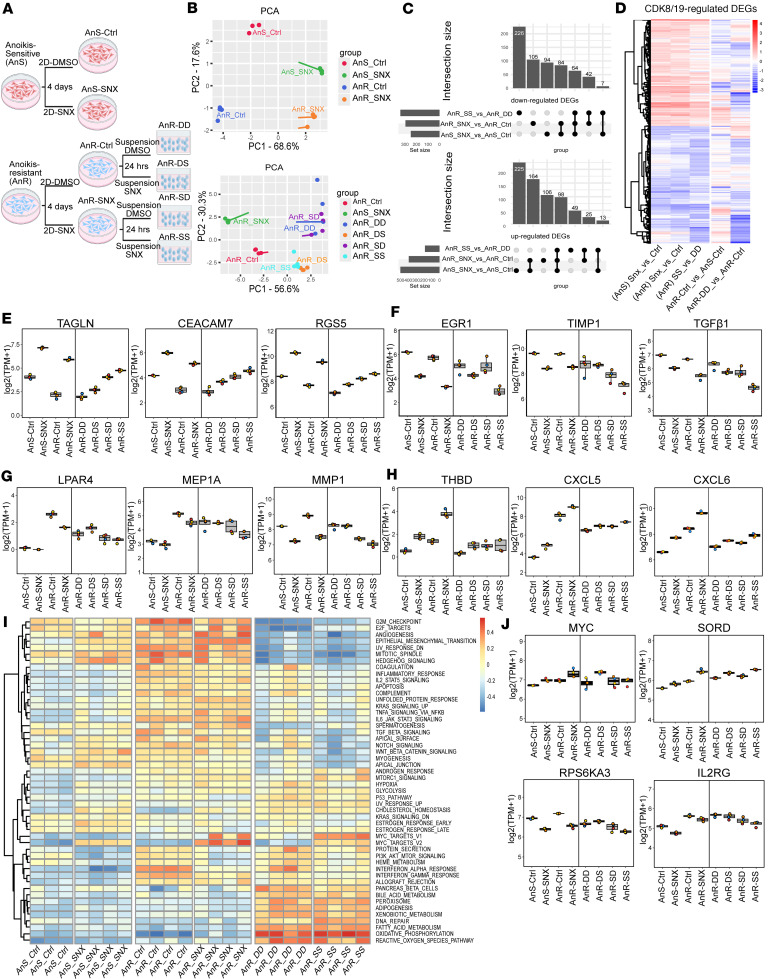
CDK8/19 inhibition induces bidirectional transcriptional reprogramming in anoikis-sensitive and -resistant cells. (**A**) Schematic of RNA-seq design; OV90 AnS and AnR cells treated with DMSO or SNX631 for 4 days in 2D; AnR cells further cultured in suspension ± SNX631. (**B**) Principal component analysis (PCA) of AnS vs. AnR transcriptomic profiles in 2D (top) and AnR cells in 2D vs. suspension (bottom). (**C**) UpSet plot of SNX631-regulated DEGs (FDR < 0.01, FC > 1.5) across 3 comparisons: AnS in 2D (AnS-SNX vs. AnS-Ctrl), AnR in 2D (AnR-SNX vs. AnR-Ctrl), and AnR in suspension (AnR-SS vs. AnR-DD). (**D**) Heatmap of all SNX631-regulated DEGs; columns 1–3: SNX631 response; column 4 (AnR-Ctrl vs. AnS-Ctrl): anoikis resistance-associated DEGs; column 5: acute anoikis response DEGs (AnR-DD vs. AnR-Ctrl). (**E** and **F**) Expression (transcripts per million) of representative DEGs upregulated (**E**) or downregulated (**F**) by CDK8/19 inhibition in AnR cells in suspension (AnR-SS vs. AnR-DD). (**G** and **H**) Expression of genes showing bidirectional regulation: reversed (**G**) or enhanced (**H**) by CDK8/19 inhibition. (**I**) GSVA heatmap of hallmark pathway NES clustered by Euclidean distance. (**J**) Expression of representative MYC targets V1 and PI3K/AKT/mTOR pathway genes.

To investigate the transcriptional basis for CDK8/19 inhibition’s effects on restoring anoikis sensitivity in AnR cells, we performed RNA-seq analyses on both anoikis-sensitive (AnS) and adapted anoikis-resistant (AnR) OV90 cells following 4 days of SNX631 treatment under standard 2D culture conditions. Since AnR cells showed enhanced anoikis susceptibility to SNX631 (Figure 7D), we also profiled AnR cells under suspension culture conditions, with or without SNX631 treatment, to capture the transcriptional response of AnR cells to anoikis stress (schematic, Figure 8A).

Principal component analysis (PCA) of the RNA-seq data revealed a clear separation between AnS and AnR cells along both PC1 (68.6% variance) and PC2 (17.6% variance), with SNX631 treatment shifting the transcriptomes of both cell types in a similar direction. Notably, the transcriptional changes induced by CDK8/19 inhibition on PC1 were opposite to those associated with the development of anoikis resistance (Figure 8B). In AnR cells, transitioning from 2D to suspension culture (AnR-DD vs. AnR-Ctrl) also led to a pronounced shift along PC1 (56.6% variance), consistent with transcriptional changes in response to anoikis stress. This shift was partially reversed by SNX631 treatment in 3D culture, in the same direction as the effect of CDK8/19 inhibition under 2D conditions (Figure 8B).

Differential expression analysis (cutoffs: fold-change >1.5, FDR < 0.01) identified 749 DEGs (500 upregulated, 249 downregulated, Supplemental Data 1) in AnS cells treated in 2D, 688 DEGs (393 up, 295 down, Supplemental Data 2) in AnR cells treated in 2D, and 524 DEGs (185 up, 339 down, Supplemental Data 3) in AnR cells treated in 3D. UpSet analysis (Figure 8C) showed that 497 genes were shared between at least 2 of these conditions, suggesting that CDK8/19 regulates a core set of genes regardless of culture conditions or anoikis resistance status. Heatmap analysis of the union of all SNX631-regulated DEGs (from all 3 comparisons) further highlighted the consistent transcriptional response induced by CDK8/19 inhibition across the different OV90 cell states (Figure 8D). Remarkably, many of these SNX631-responsive genes were also altered during the acquisition/development of anoikis resistance (AnR-Ctrl vs. AnS-Ctrl) or upon exposure to anoikis stress (AnR-DD vs. AnR-Ctrl), suggesting that CDK8/19 activity is essential for maintaining the balanced transcriptional programs that enable aggressive OC cells to survive under detachment stress.

Examination of a subset of the top DEGs upregulated or downregulated by CDK8/19 inhibition in AnR cells under suspension conditions (AnR-SS vs. AnR-DD, Figure 8, E and F) revealed upregulated genes including *TAGLN*, *CEACAM7*, and *RGS5*, while downregulated genes included *EGR1*, previously identified as an SNX631-responsive gene in other cellular models (35, 54, 59, 60), as well as *TIMP1* and *TGFB1*, with *TGFB1* particularly known for its role in anoikis resistance and OC metastasis (23, 61–63). Notably, most of these genes were downregulated in AnR cells, with some further decreased upon suspension culture. We also examined DEGs upregulated in AnR cells and found 2 distinct responses to CDK8/19 inhibition: Induction of some genes was reversed (e.g., *LPAR4*, *MEP1A*, *MMP1*; Figure 8G), whereas expression of others was enhanced (e.g., *THBD*, *CXCL5*, *CXCL6*; Figure 8H). Additionally, we observed that omitting SNX631 treatment from either 2D (AnR-DS) or suspension conditions (AnR-SD) diminished the transcriptional regulatory effects of CDK8/19 inhibition on most of these genes, consistent with the most significant effects on anoikis observed upon continuous treatment (Figure 7C), suggesting that sustained CDK8/19 activity is required for maintaining the dynamic transcriptional programs underlying the transcriptional plasticity. GSVA using hallmark gene sets (Figure 8I) revealed widespread pathway alterations across different cell states. Among these, several pathways upregulated in AnR cells were suppressed by CDK8/19 inhibition, including PI3K/AKT/mTOR signaling, heme metabolism, and interferon responses. Conversely, the MYC targets gene set, upregulated in AnR cells, were further increased by SNX631, indicating that CDK8/19 inhibition induces bidirectional disruption of transcriptional pathways. Representative genes illustrating these effects include *RPS6KA3* and *IL2RG* (PI3K/AKT/mTOR pathway) and *MYC* and *SORD* (MYC pathway), respectively (Figure 8J). Together, these analyses demonstrate that CDK8/19 inhibition disrupts balanced, pathway-specific transcriptional reprogramming that underlies key mechanisms of anoikis resistance.

## Discussion

We report here that OC cells can acquire anoikis resistance through nongenetic adaptation when subjected to cycles of detachment and reattachment. This acquired resistance mimics that observed in ascites-derived cells but is reversible, with cells returning to baseline sensitivity without detachment stress. Importantly, the acquired adaptive and transient anoikis resistance is sufficient to confer aggressive phenotypes, including enhanced migration, paclitaxel resistance, increased metastatic potential, metabolic reprogramming, and immune evasion. CDK8/19 mediator kinase inhibition both prevents and reverses this anoikis resistance while disrupting the balanced transcriptional program underlying such adaptation.

The cyclic detachment model recapitulates key features of i.p. OC progression, where cells repeatedly detach from tumor masses, survive in ascites, and reattach at distant sites. Although OC dissemination involves both multicellular aggregates and single cells, and vascular metastasis may occur faster than anoikis kinetics, the extended suspension exposure during i.p. spread, the dominant route in OC, makes anoikis resistance functionally relevant regardless of cluster size. The nongenetic nature of this adaptation, confirmed by whole-exome sequencing, aligns with evidence that recurrent ascites tumors contain mutations already present initially (64), indicating epigenetic/transcriptional adaptation rather than genetic evolution–driven recurrence. Clonal fluctuation analysis revealed that individual clones interchange between sensitive and resistant states, with behavioral heterogeneity exceeding that of control populations. This cellular diversity, rather than a simple 2-state model, suggests multiple cellular states that warrant future investigation. Whether resistance decay reflects selective pressure or active transcriptional overwriting by adhesion signaling cannot be distinguished by our data, though CDK8/19 dependency suggests coordinated programs beyond immediate adhesion responses.

A central finding is that transient, nonpermanent anoikis resistance is sufficient for metastatic capabilities. Adapted cells showed increased migration, consistent with the reciprocal relationship between motility and anoikis resistance (49, 65). Although chemoresistance was specific to paclitaxel in standard assays, testing under conditions mimicking anoikis selection may reveal additional resistance mechanisms to cisplatin and doxorubicin. Metabolically, adapted cells exhibited increased oxygen consumption, ATP production, SRC, and glycolysis, indicating metabolic flexibility. These could present therapeutic opportunities through dual metabolic targeting that combines OXPHOS inhibitors (which AnR cells showed heightened sensitivity to) with glycolytic inhibitors (targeting LDH or MCT) or pH regulation disruptors (CAIX inhibitors), which could eliminate compensatory metabolic escape and selectively stress these adapted cells. Anoikis-adapted cells also showed reduced MHCI and TAP1 expression with functional immune evasion. These multiple adaptations highlight why preventing anoikis resistance development, rather than targeting a single established resistance mechanism, may be more effective. CDK8/19 inhibition could both prevent and reverse these phenotypes, suggesting this as a more tractable therapeutic strategy. Whether anoikis resistance fully overlaps with broader death resistance remains to be determined, though the enhanced paclitaxel chemoresistance alongside sensitivity to OXPHOS inhibitors suggest context specificity rather than pan-resistance.

Moreover, while there was consistent acquisition of metastatic phenotypes across models, the timing of pathway changes varied between cell lines. CAOV3 cells, which are more sensitive to detachment stress and carry different mutations than OV90, reverted to sensitivity faster. Whether baseline sensitivity or mutational background drives these timing differences remains to be determined. Regardless, the dependency on transcriptional reprogramming was evident across all models through marked sensitivity to CDK8/19 inhibition.

CDK8 and CDK19 are alternative enzymatic components of the CDK module regulating the transcriptional mediator complex, dispensable for basal transcription, but critical for signal-induced transcriptional responses (35, 36, 66). Consistent with this selective role, SNX631 at 500 nM, sufficient for complete kinase inhibition (67), had minimal effects on steady-state growth yet potently blocked anoikis resistance development across multiple models. This selective vulnerability aligns with studies showing CDK8/19 inhibition prevents adaptive drug resistance (32, 54). Strikingly, SNX631 not only prevented anoikis resistance but also resensitized adapted cells, with maximal effects achieved through combined pretreatment and acute exposure during suspension stress.

Transcriptomic analysis revealed that resensitization involves reprogramming rather than simple reversal. CDK8/19 inhibition induced bidirectional effects with about half the changes shared between sensitive and resistant cells, representing a core CDK8/19-dependent program. Some resistance pathways were reversed and others paradoxically enhanced, reflecting CDK8/19’s established role as both a positive and negative transcriptional regulator (35) and as a molecular rheostat maintaining the transcriptional balance required for anoikis resistance. Since CDK8/19 RNA levels were unchanged during adaptation, the heightened vulnerability of adapted cells likely reflects transcriptional addiction to CDK8/19-dependent stress response programs. Notably, this dependency persists even under active suspension conditions where anoikis resistance is functionally critical, suggesting that repeated detachment stress creates lasting reliance on these transcriptional mechanisms.

The selective targeting of stress-induced transcriptional plasticity while sparing basal transcription makes CDK8/19 an attractive therapeutic target. Prior work established a correlation between CDK8/19 expression and chemotherapy failure (60) and ovarian clear cell carcinoma (OCCC) metastasis (38) and demonstrated the chemosensitizing effect of CDK8/19 inhibition in OCCC (38). Our present findings extend these results and provide their mechanistic underpinning by linking CDK8/19 activity to anoikis resistance and stress-induced transcriptional plasticity underlying metastatic adaptation. Although CDK8/19 expression correlates with patient survival in OC subtypes (38, 60, 68), our findings suggest that therapeutic efficacy depends on targeting transcriptional dependencies rather than expression levels. Genes upregulated in anoikis resistance that are reversed by SNX631 could serve as biomarkers for patient selection. Given OC’s extraordinary adaptability during i.p. dissemination and the limited targeted therapy options, CDK8/19 inhibition represents an opportunity to both prevent and reverse metastatic adaptation.

## Methods

### Sex as a biological variable.

Our study examined only female mice because OC is a disease that exclusively affects females. The findings from this study are expected to be relevant to females.

### Cell lines and culture conditions.

Ovarian cancer cell lines (OV90, CAOV3, OVCAR3, OVCAR4, OVCAR5, OVCAR10, SK-OV3, OVCA420, HEY, HEYA8, TYK-nu), patient-derived lines (P76, P151, P201, P210, EOC15), immortalized fallopian tube epithelial cells (FT282), immortalized ovarian surface epithelial cells (IOSE141), murine OC cells (ID8-EMD), and HEK293 cells were maintained in media as detailed in Supplemental Methods and Supplemental Table 1. Cell lines were obtained from ATCC, from the NIH NCI60 panel, or as gifts from Susan Murphy (Duke University, Durham, North Carolina, USA) and Amir Jazaeri (MD Anderson Cancer Center, Houston, Texas, USA). All cell lines were authenticated (UAB Heflin Center for Genomic Sciences) and mycoplasma tested (LookOut Mycoplasma PCR Detection Kit, MilliporeSigma, catalog MP0035-1KT). Cells were maintained at 37°C in 5% CO_2_. All reagents, antibodies, and resources are listed in Supplemental Tables 2–6 (see Supplemental Methods).

### Patient and mouse ascites-derived cells.

Patient ascites cells were established as previously described (19, 23). For immortalization, cells were transduced with hTERT and SV40 large T-antigen lentivirus (GenTarget, catalog LVP1130-Puro-PBS and LVP016-Hygro) and selected with puromycin. See Supplemental Methods for details.

### Cell viability and apoptosis assays.

Viability was assessed by trypan blue exclusion, SRB assay, or LIVE/DEAD Viability/Cytotoxicity Kit (Thermo Fisher Scientific, catalog L3224) with confocal imaging. Apoptosis was measured by flow cytometry using annexin V/PI staining (eBioscience, catalog 88-8005-74) on a BD LSRFortessa and analyzed in FlowJo 10.8.1. Proliferation was assessed by Ki67 immunofluorescence (Cell Signaling Technology, catalog 9449, 1:450). IC_50_ values were determined using SRB assay with 8-point dilution series and calculated by nonlinear regression in GraphPad Prism. Additional details of protocols for each are in Supplemental Methods.

### Cyclic cell culture/anoikis resistance model.

For suspension culture, 250,000 cells were cultured on poly-HEMA–coated, 6-well plates for 24 hours. After dissociation with 10× trypsin-EDTA (MilliporeSigma), viability was assessed by trypan blue exclusion using a Countess II FL Automated Cell Counter (Thermo Fisher Scientific, catalog AMQAF1000). To generate AnR cells, AnS cells underwent 7–9 cycles of 24-hour suspension culture followed by expansion under standard 2D conditions until 90% confluent. For memory/reversion studies, AnR cells were maintained in 2D culture for 9–11 passages, with suspension survival assessed at each passage. Single-cell cloning was performed by serial dilution. Additional details are provided in Supplemental Methods.

### Mitochondrial respiration.

OCR was measured using the Seahorse XF96 Mito Stress Test (Agilent; UAB Bioanalytical Redox Biology Core) with sequential injections of oligomycin (1.5 μM), FCCP (0.9–1.8 μM), and antimycin A/rotenone (0.5 μM each). For OXPHOS inhibitor sensitivity, cells were treated with oligomycin (1.5 μM) or IM156 (15–25 μM) for 72 hours in suspension, followed by annexin V/PI staining. See Supplemental Methods for complete protocols.

### Immune-mediated tumor-killing assay.

CD8^+^ T cells were isolated from healthy donor PBMCs (Precision for Medicine) using the Miltenyi Biotec CD8^+^ isolation kit (catalog 130-096-495), activated with CD3/CD28 Dynabeads (Gibco, catalog 11161D) for 48 hours, and cocultured with tumor cells at 10:1 ratio. Viability was assessed by trypan blue exclusion and apoptosis by CC3 immunofluorescence (Cell Signaling Technology, catalog 9661T). See Supplemental Methods for details.

### CDK8/19 inhibitor treatments.

SNX631 and THZ1 were obtained from Senex Biotechnology and MedChemExpress, respectively. For prevention studies, cells were cultured through attachment-detachment cycles in the presence of 500 nM SNX631 or DMSO vehicle. For reversal studies, AnR cells were pretreated with 500 nM SNX631 for 96 hours under 2D conditions before suspension challenge. CDK8/19 kinase inhibition was confirmed by immunoblotting for phospho-STAT1 (Cell Signaling Technology, catalog 9177S). See Supplemental Methods for detailed protocols.

### Transwell fibronectin migration.

Transwell migration was performed using 8 μm pore membranes (Greiner Bio-One, catalog 662638) coated with fibronectin (10 μg/mL). Cells in serum-free medium were placed in the upper chamber with complete medium in the lower chamber. After 6–24 hours (cell line dependent, Supplemental Table 7), migrated cells were fixed, stained with crystal violet, imaged on an EVOS M7000 microscope (Thermo Fisher Scientific, catalog AMF7000), and quantified using ImageJ (NIH).

### Animal studies.

Female NSG (NOD.Cg-*Prkdc^scid^ Il2rg^tm1Wjl^*/SzJ), SCID (NOD.Cg-*Prkdc^scid^*/J), and C57BL/6J mice (8 weeks old, Jackson Laboratories) were housed in pathogen-free conditions. For anoikis resistance model studies, SCID mice received i.p. injection of 5 × 10^6^ OV90-LUC-GFP cells (AnS or AnR) and were monitored by IVIS BLI (150 μg/mL d-luciferin) every 10 days until day 39–40. For ID8 syngeneic studies, C57BL/6J mice received 10 × 10^6^ ID8-EMD cells (AnS or AnR) i.p. and were monitored for 11.5 weeks. For SNX631-6 efficacy studies, NSG mice received OV90-LUC-GFP cells (5 × 10^6^ i.p.) and were randomized to control diet or SNX631-6–medicated diet (350 ppm, ~30–50 mg/kg daily; Senex Biotechnology). Terminal analyses included ascites volume, tumor weight, omental weight, and lung bioluminescence. See Supplemental Methods for additional details

### RNA sequencing and analysis.

Total RNA was isolated using TRIzol (Thermo Fisher Scientific, catalog 15596018)/chloroform (Thermo Fisher Scientific, catalog ICN19400291) extraction and quality-validated on Bioanalyzer (Agilent). Libraries were prepared using NEBNext Ultra RNA Library Prep Kit (New England Biolabs) and sequenced on Illumina NovaSeq 6000. Reads were aligned to GRCh38 using STAR, and counts were generated with featureCounts. Differential expression analysis was performed using DESeq2 with FDR < 0.05 and |L2FC| ≥ 1 cutoffs. Pathway analysis used GSVA with MSigDB Hallmark gene sets. Heatmaps were generated with ComplexHeatmap. UpSet plots were created using ComplexUpset R package v 1.3.3 (https://cran.r-project.org/web/packages/ComplexUpset/index.html and ref. 69). Complete bioinformatics pipelines are detailed in Supplemental Methods.

### Whole-exome analysis.

DNA was isolated using DNeasy Blood and Tissue Kit (QIAGEN, catalog 69504). WES was performed by Novogene using Agilent SureSelect Human All Exon V6 with >159× median coverage on NovaSeq 6000. Variant calling followed GATK best practices using HaplotypeCaller and Mutect2. Analysis was performed with MAFtools (70). See Supplemental Methods for details.

### Semiquantitative RT-qPCR.

RNA was extracted with TRIzol/chloroform, reverse-transcribed using iScript Reverse Transcription Supermix (Bio-Rad, catalog 11708840), and amplified with iTaq Universal SYBR Green Supermix (Bio-Rad, catalog 1725125). Expression was normalized to RPL13A, HPRT, or GAPDH. Primer sequences are listed in Supplemental Table 6.

### Statistics.

Data were analyzed using GraphPad Prism 10 and expressed as mean ± SEM. Statistical significance was defined as *P* < 0.05. Two-tailed unpaired *t* tests were used for comparisons between 2 groups. One-way ANOVA with Tukey’s multiple comparison was used for single-variable comparisons across multiple groups; 2-way ANOVA with Tukey’s multiple comparison was used for 2-variable comparisons. Experiments represent at least 3 independent biological trials with multiple technical replicates unless otherwise indicated.

### Study approval.

All animal studies were reviewed and approved by the Institutional Animal Care and Use Committee at the University of Alabama at Birmingham. Patient ascites samples were obtained with prior approval from the Penn State College of Medicine Institutional Review Board, Hershey, Pennsylvania, USA; written informed consent was obtained from all participants.

### Data availability.

RNA-seq data are available at NCBI GEO under accession numbers GSE241546 and GSE309005. Analysis code is available at https://github.com/page22emily/RNAseq_Anoikis; commit ID 6f291be. Supporting data values for all figures are provided in the supplemental Supporting Data Values file.

## Author contributions

MM and RR contributed equally as co–first authors; authorship order was determined by mutual agreement. KM, MM, AS, and EVB were responsible for conceptualization. MM, RR, AK, LQM, SS, FM, and MC were responsible for investigation. KM, MM, RR, EFP, NYL, SS, NH, MKJ, LI, EW, AS, IBR, EVB, and MC were responsible for analysis. KM, NH, AS, IBR, MKJ, and EW were responsible for resources/supervision. MM, RR, IBR, MC, and KM were responsible for writing the original draft. All authors were responsible for review and editing. KM was responsible for funding acquisition and project administration. The first coauthors may list their name first on publication lists, such as CVs, to demonstrate their equal contribution.

## Funding support

This work is the result of NIH funding, in whole or in part, and is subject to the NIH Public Access Policy. Through acceptance of this federal funding, the NIH has been given a right to make the work publicly available in PubMed Central.

- NIH grants R01CA230628 (KM and NH), R35GM148351 (AS), R43CA271996, and R01CA266027 (EVB and MC).
- Norma Livingston Ovarian Cancer Foundation (KM).
- University of South Carolina COBRE Center for Targeted Therapeutics Functional Genomics, Microscopy and Flow Cytometry, and Drug Design and Synthesis Cores (P20GM109091).
- UAB shared resources: Biological Data Science Core (RRID:SCR_021766), Flow Cytometry Core (AI027767), O’Neal Comprehensive Cancer Center (P30CA013148), Preclinical Imaging Shared Facility (P30CA013148, 1S10OD021697), High Resolution Imaging Facility, and Bio-Analytical Redox Biology Core including Melissa J. Sammy, PhD (P30DK079626, P30DK056336, UL1TR003096), and Pathology Core Research Lab.

## Supplementary Material

Supplemental data

Supplemental data set 1

Supplemental data set 2

Supplemental data set 3

Unedited blot and gel images

Supporting data values

## Version 1 01/15/2026

In-Press Preview

## Version 2 02/23/2026

Electronic publication
