# Supplementary material for: Anoikis resistance and metastasis of ovarian cancer can be overcome by CDK8/19 mediator kinase inhibition
Source: JCI Insight. 2026 Jan 15;11(4):e192113. doi: 10.1172/jci.insight.192113 (PMC12956021; doi:10.1172/jci.insight.192113)
Supplement: Supplemental data [file jciinsight-11-192113-s201.pdf]

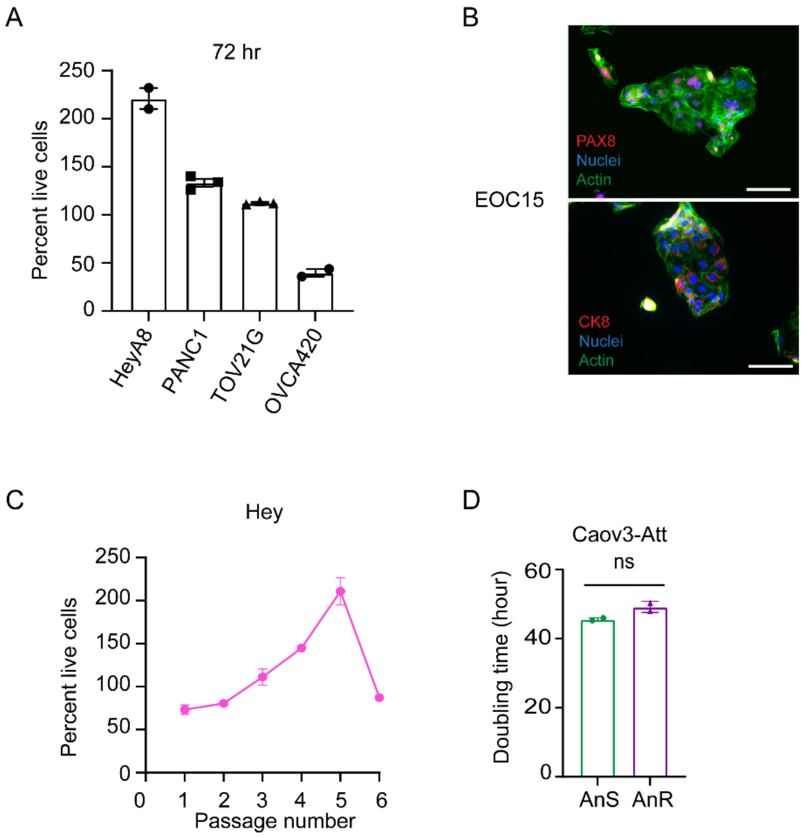

Supp Figure 1:

(A) Percent live cells in suspension of cell lines HeyA8, PANC1, TOV21G, and OVCA420 measured by trypan blue staining following 72 hours of incubation in suspension culture (n = 2-3 replicates/cell line). **(B)** Representative immunofluorescence images of F-actin (green), PAX8, and CK8 (red) from EOC15 primary cells, Scale bar: 100 µm. **(C)** Percent live cells in suspension of Hey cells following cyclic model of gain and loss of attachment from Fig 1B. Live cells were measured by trypan blue staining after 24 hours in suspension and plotted as percent relative to initial plating number (n = 2-3 replicates). Live cells were measured by trypan blue staining after 24 hours in suspension and plotted as percent relative to initial plating number (n = 3 replicates) **(D)** Doubling time of parental AnS CAOV3 cells or AnR CAOV3 AnR derivative in attached (Att) growth conditions calculated over a 10-day period using an SRB assay. All data are mean ± SEM. ns $p > 0.05$, * $p < 0.05$, ** $p < 0.01$, *** $p < 0.001$, Two-way ANOVA followed by Tukey's multiple comparison or unpaired t-test.

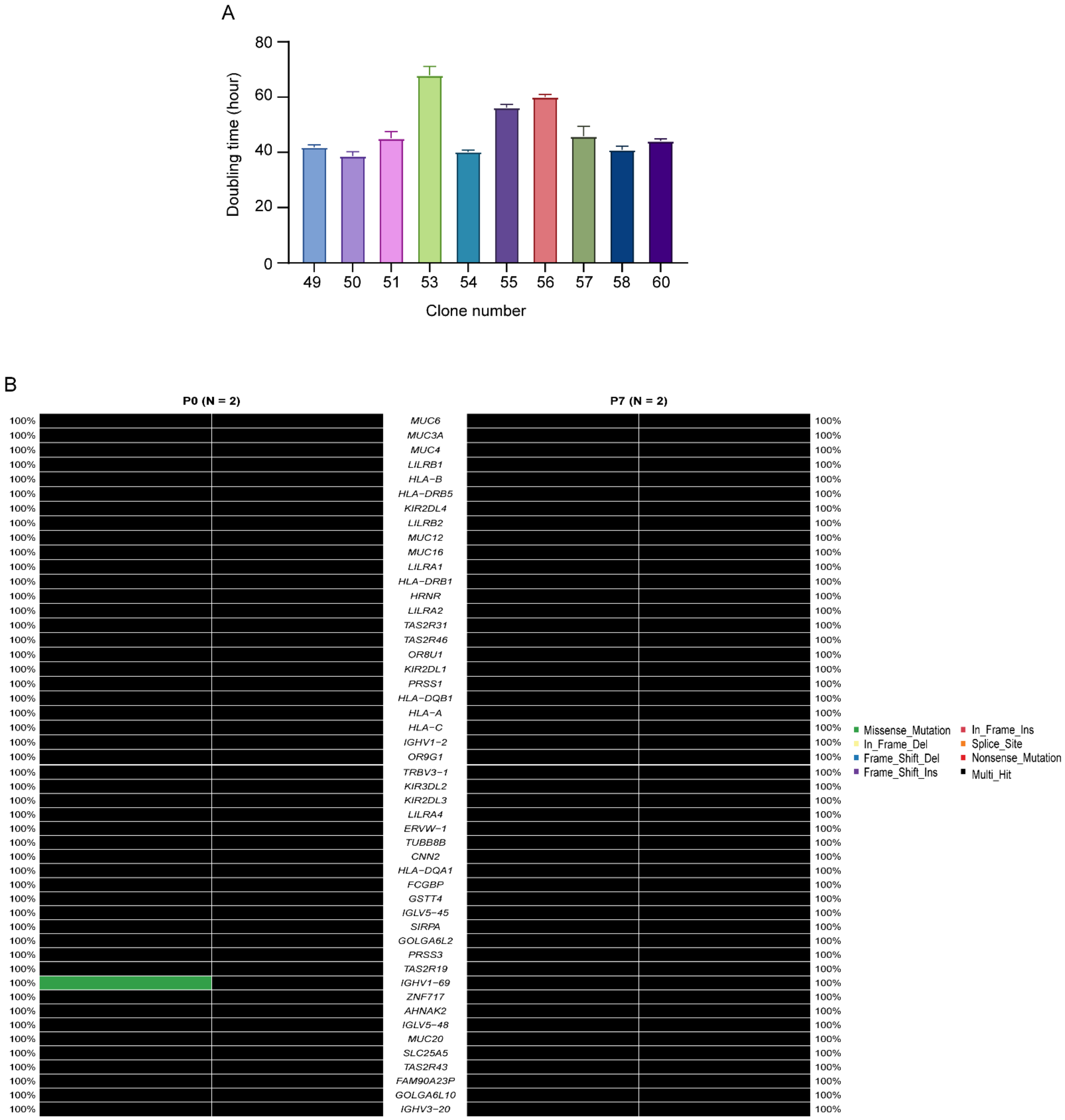
A
Doubling time (hour)
0
20
40
60
80
49
50
51
53
54
55
56
57
58
60
Clone number
B
P0 (N = 2)
P7 (N = 2)
MUC6
MUC3A
MUC4
LILRB1
HLA−B
HLA−DRB5
KIR2DL4
LILRB2
MUC12
MUC16
LILRA1
HLA−DRB1
HRNR
LILRA2
TAS2R31
TAS2R46
OR8U1
KIR2DL1
PRSS1
HLA−DQB1
HLA−A
HLA−C
IGHV1−2
OR9G1
TRBV3−1
KIR3DL2
KIR2DL3
LILRA4
ERVW−1
TUBB8B
CNN2
HLA−DQA1
FCGBP
GSTT4
IGLV5−45
SIRPA
GOLGA6L2
PRSS3
TAS2R19
IGHV1−69
ZNF717
AHNAK2
IGLV5−48
MUC20
SLC25A5
TAS2R43
FAM90A23P
GOLGA6L10
IGHV3−20
100%
Missense_Mutation
In_Frame_Del
Frame_Shift_Del
Frame_Shift_Ins
In_Frame_Ins
Splice_Site
Nonsense_Mutation
Multi_Hit

Supp Figure 2:

(A). Doubling time of indicated clones (randomly chosen) measured by an SRB assay every 24- 48 hours over a 7-day period. **(B)**. Oncoplot of the top 50 mutated genes based on the total number of mutations present across 2 time points, P0 (left) and P7 (right).

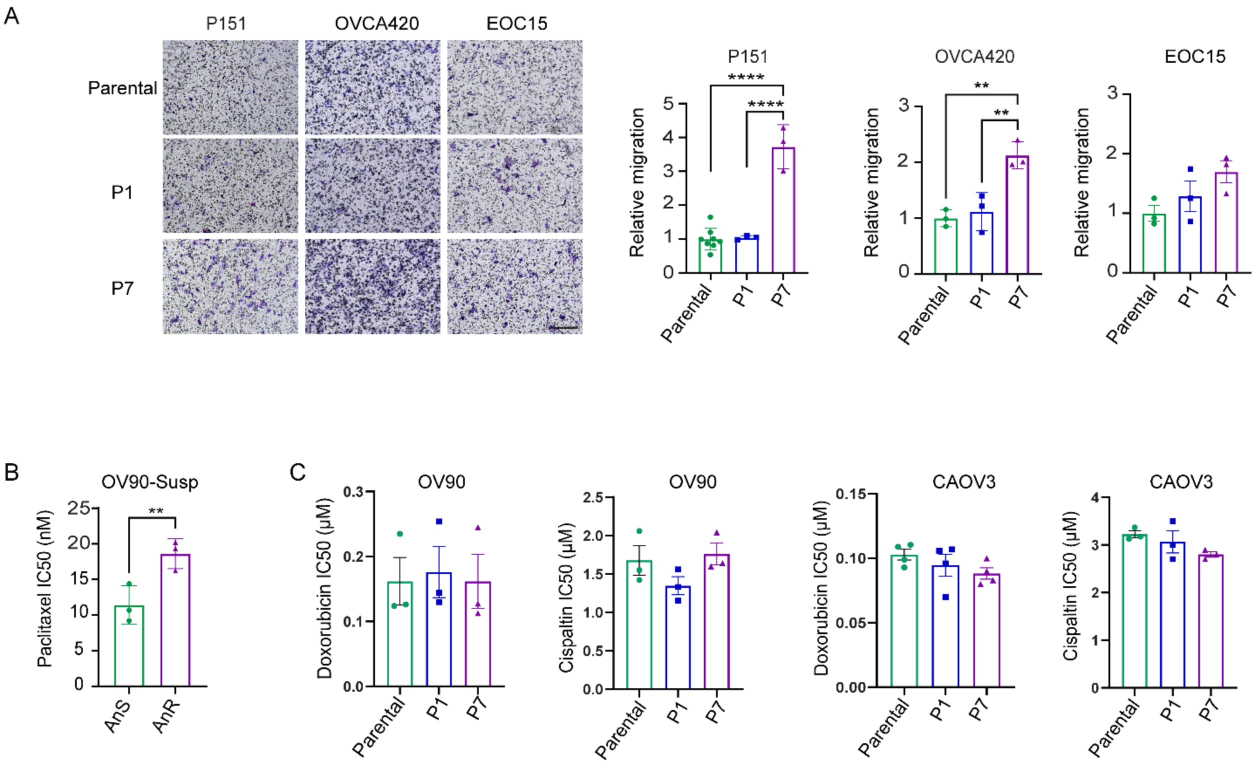

Supp Figure 3:

(A). Representative images (left) and quantitation (right) of indicated AnS parental, P1 and P7 (AnR) cells on fibronectin coated transwell filters after 24 hrs. of migration (n=3). Scale bar: 200µm. **(B).** IC50 of paclitaxel in OV90 cell lines determined under suspension culture conditions over a period of 72 hrs. using CellTiter-Glo 3D cell viability assay. **(C)** IC50 of indicated parental, P1 (cells expanded after one 24 hr exposure to suspension culture) and P7 (AnR) cells to doxorubicin and cisplatin under steady attached conditions over a period of 72 hrs. using an SRB assay (n=3). Data are Mean ± SEM, ns $p > 0.05$. One way ANOVA followed by Tukey's multiple comparison for A,C and unpaired t test for B.

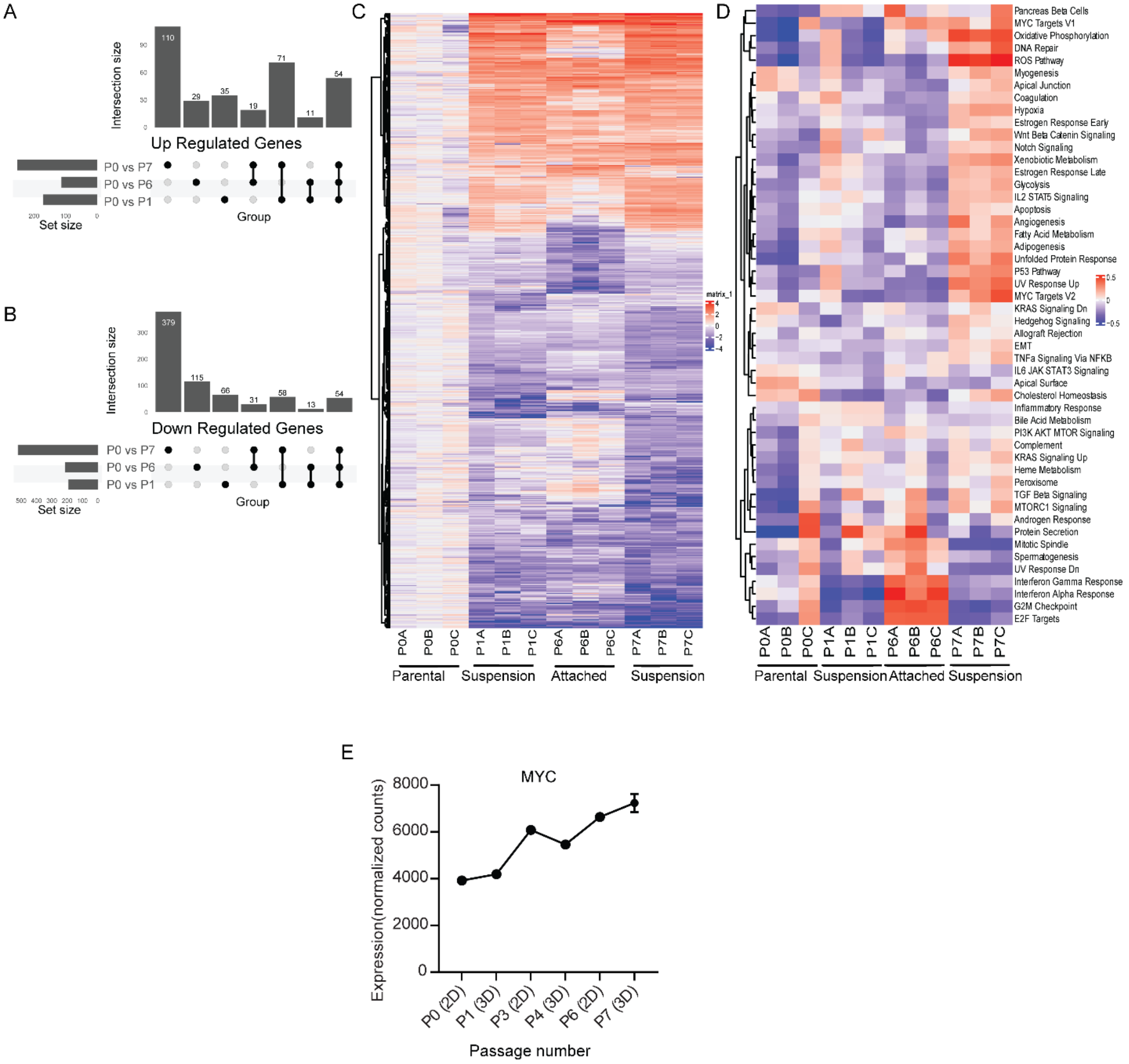

Supp Figure 4:

(A). UpSet plots indicating the number of up regulated genes (p value < 0.05 and L2FC >1.5) and **(B)** down regulated genes (p value < 0.05 and L2FC < -1.5) in CAOV3 across the time point

comparisons P0 vs P1, P0 vs P6, and P0 vs P7. **(C)** Heatmap for the individual CAOV3 samples using Log2FC as calculated for individual biological replicates for parental P0, (P0A-C), P1 (P1A- C), P6 (P6A-C), and P7 (P7A-C). Respective attached and suspension time points are indicated. Heatmap generated by clustering analysis of DEGs across samples (1041 genes). The Log2 fold changes of each gene were clustered based on euclidean distance. **(D).** Heatmaps for the individual CAOV3 samples using GSVA normalized enrichment scores for passage 0 (P0A-C), 1 (P1A-C), 6 (P6A-C), and 7 (P7A-C). The hallmarks were clustered based on euclidean distance. **(E)** c- MYC expression (normalized count from RNA sequencing) in indicated OV90 cells cultured using model of cyclic gain and loss of attachment as in Fig 1B.

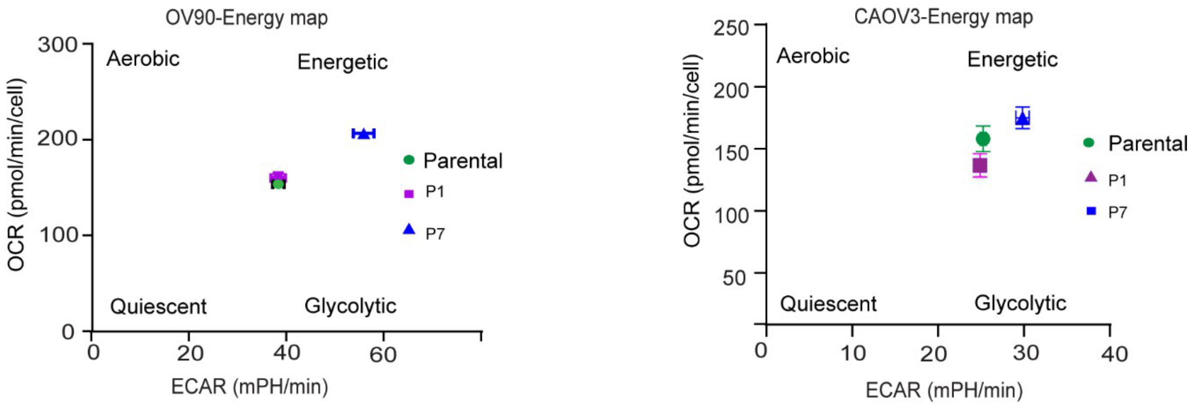

Supp Figure 5:

Energy map of indicated OV90 and CAOV3 cells under attached conditions measured using the mito stress assay on seahorse XFe96 extracellular flux analyzer.

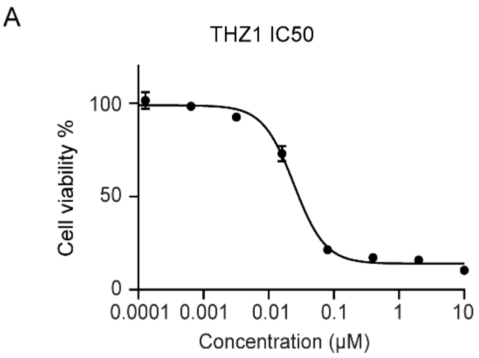

Cell line	THZ1 Mean IC50 (µM)
OV90	0.034

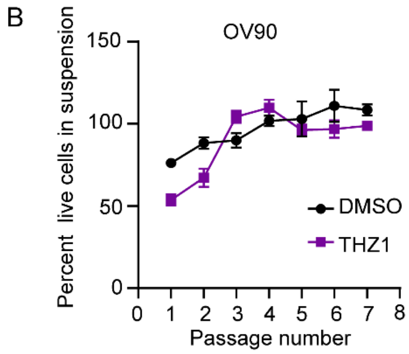

Supp. Figure 6:

(A) IC50 of OV90 cells assessed after 3 days of incubation with either vehicle or THZ1 using an SRB assay. Adjacent table with calculation of IC50 from A. **(B)** Percent survival of OV90 cells measured by trypan blue staining after 24 hours in suspension following cycles of gain and loss of attachment as in Fig 1B, either with vehicle (DMSO) or in the presence of THZ1 (n = 3).

SUPPLEMENTARY METHODS

Cell lines and culture conditions

DMEM-based cultures:

CAOV3, P76, P151, P201, P210, and HEK293 cells were cultured in DMEM containing high glucose, L-glutamine, and sodium pyruvate, supplemented with 10% FBS.

TYK-nu cells were cultured in DMEM supplemented with 1% MEM non-essential amino acids and 1% MEM vitamins.

ID8-EMD murine ovarian cancer cells were cultured in DMEM with 4% FBS, 5 µg/mL insulin, 5 µg/mL transferrin, and 5 ng/mL sodium selenite.

Mixed media cultures:

FT282 immortalized fallopian tube epithelial cells were cultured in a 1:1 mixture of DMEM and F12 medium supplemented with 10% FBS and 2 mM L-glutamine.

OV90 and EOC15 cells were cultured in a 1:1 mixture of MCDB 105 and Medium 199 supplemented with 15% FBS.

IOSE141 immortalized ovarian surface epithelial cells were cultured in a 1:1 mixture of MCDB 105 and Medium 199 supplemented with 10% FBS.

RPMI-based cultures:

SK-OV3, OVCA420, HEY, HEYA8, and OVCAR10 cells were cultured in RPMI (containing L-glutamine) supplemented with 10% FBS.

OVCAR3 cells were cultured in RPMI supplemented with 20% FBS.

OVCAR4 and OVCAR5 cells were cultured in RPMI supplemented with 10% FBS, 2 mM glutamine, and 0.25 U/mL insulin.

Patient and mouse ascites-derived cells

For processing, patient-derived ascites fluid (50 ml) or mouse ascites fluid (collected post-OV90 or ID8 i.p. injection at endpoint) was centrifuged at 4000 rpm for 20 minutes. RBC lysis buffer was added to the cell pellet at a 10:1 volume ratio and incubated for 10 minutes at room

temperature. PBS was added and cells were centrifuged at 300g for 10 minutes. Cells were resuspended in appropriate culture medium, counted using the Countess II FL, and cultured in poly-HEMA-coated plates (250,000 cells per well) for 2 weeks with gentle disaggregation after one week. Cells were then transitioned to 2D culture for expansion. For immortalization, cells (50,000 per well) were transduced with hTERT (GenTarget, cat# LVP1130-Puro-PBS) and SV40 large T-antigen (GenTarget, cat# LVP016-Hygro) lentiviral constructs at MOI 15 in the presence of polybrene (10 μg/ml) for 24 hours. After 48 hours of recovery, cells were selected with puromycin (5 μg/ml) for 48 hours.

Poly-HEMA coating for suspension culture

6-well tissue culture plates were coated with poly-HEMA (Sigma Aldrich, cat# P3932-25G) prepared as a 2% solution in 95% ethanol. One milliliter of poly-HEMA solution was added to each well and plates were allowed to dry at 50°C overnight. Dried plates were UV-irradiated for 1 hour before use. Coated plates were either used immediately for experiments or wrapped with Parafilm and stored at 4°C for later use.

Viability and Apoptosis Assays

Viability assessment of cell aggregates in suspension (multiple spheroids or cell aggregates)

Cells were cultured in 100 mm plates until reaching approximately 90% confluency, then trypsinized and counted using Trypan blue exclusion with a Countess II FL Automated Cell Counter (Thermo Fisher). For suspension culture, 250,000 cells were seeded in poly-HEMA-coated 6-well plates in 3 ml of complete growth medium per well and incubated for 24 hours. To assess viability, cells from each well were collected in a 15 ml tube and centrifuged at 1200 rpm for 5 minutes. The medium was removed and 100 μl of 10× Trypsin-EDTA (Millipore Sigma, cat# T4174-100ML) was added to dissociate cell aggregates. Cells were incubated at 37°C for 5-20 minutes depending on cell line and spheroid compactness. After dissociation, 400 μl of complete medium was added to neutralize trypsin, and live cell counts were determined by Trypan blue exclusion using the Countess II FL Automated Cell Counter.

Viability assessment of single spheroids

To generate single spheroids, cells (1,000 OV90 or 2,500 CAOV3) were transferred to ultra-low attachment U-bottom black-wall 96-well plates (Corning, cat# 4515) in 200 µl medium. Plates were centrifuged at 1200 rpm for 2 minutes to promote cell aggregation and then incubated for 24 hours. For live/dead imaging, the LIVE/DEAD Viability/Cytotoxicity Kit for mammalian cells (Fisher Scientific, cat# L3224) was used. Green-fluorescent Calcein-AM and red-fluorescent ethidium homodimer-1 were diluted in HBSS (Hanks' Balanced Salt Solution) or PBS to 4 µM and 8 µM respectively. Half of the culture medium (100 µl) was carefully removed from each well and replaced with 100 µl of HBSS containing the dyes, achieving final concentrations of 2 µM Calcein-AM and 4 µM ethidium homodimer-1. After 90 minutes of incubation, spheroids were washed three times by carefully removing 100 µl of dye solution and adding 100 µl of fresh HBSS, taking care not to disturb the spheroids. Imaging was performed using a Nikon TE2000 inverted confocal microscope at UAB's High Resolution Imaging Facility, and images were analyzed in ImageJ.

Flow cytometry-based apoptosis assay

Parental and AnR OV90 and CAOV3 cells were cultured in suspension for 24 hours as described above. Cells were dissociated with Trypsin-EDTA for 5-10 minutes to produce a single-cell suspension. Medium was added to neutralize trypsin. Cells (500,000-1,000,000) were transferred to a 1.5 ml microcentrifuge tube and washed twice with cold PBS. Cells were stained with Annexin V (BioLegend, cat# 640906) and propidium iodide (Invitrogen, cat# 00-6990-42) according to the manufacturer's protocol from the Annexin V Apoptosis Detection Kit (eBioscience, cat# 88-8005-74). Flow cytometry was performed using a BD LSRFortessa (Europa) at the UAB Flow Cytometry Core. Data analysis was carried out using FlowJo version 10.8.1.

Ki-67 immunofluorescence staining in suspension

Suspension cells (1×10^6) were collected after 24 hours and centrifuged at 1200 rpm for 5 minutes. Cells were washed in cold PBS, fixed with 4% paraformaldehyde (pH 7.4) for 15 minutes, quenched with 10 mM NH_4Cl for 5 minutes, permeabilized with 0.3% Triton X-100 for 10 minutes, and blocked with 5% BSA for 1 hour at room temperature. Cells were incubated

with Ki-67 antibody (Cell Signaling, cat# 9449, clone 8D5, 1:450 dilution in 3% BSA) overnight at 4°C, followed by incubation with Alexa Fluor-conjugated secondary antibody (Invitrogen, cat# A-21125, 1:500 dilution) for 1 hour and DAPI (1:2000 dilution) for 10 minutes. Cells were cytospun onto glass slides at 800 rpm for 5 minutes and mounted with ProLong Gold Antifade Mountant (Invitrogen, cat# P36930). Images were captured using a Nikon TE2000 confocal microscope and analyzed in ImageJ. Ki-67 corrected total cell fluorescence (CTCF = integrated density minus area × background) was normalized to DAPI CTCF.

Anoikis Resistance Model

Cyclic cell culture for generating anoikis-resistant cells

To model the development of anoikis resistance, 250,000 cells were cultured in poly-HEMA-coated 6-well plates for 24 hours. Cell number and dish size were kept constant across all passages to ensure reproducible conditions. After 24 hours in suspension, cells were centrifuged at 1200 rpm for 5 minutes and viability was measured as described under 'Viability assessment of cell aggregates in suspension.' Surviving cells were then plated into standard tissue culture conditions in 60 mm dishes and allowed to grow until reaching 90% confluency. The medium was changed every 3-4 days during attached growth. This cycle of suspension stress followed by attached recovery was repeated for 6-8 additional cycles depending on the cell line (total 7-9 passages), generating anoikis-resistant (AnR) derivatives from initially anoikis-sensitive (AnS) parental cells.

Memory/reversion studies

To assess the stability of acquired anoikis resistance, adapted AnR OV90 and CAOV3 cells from suspension culture were seeded into 60 mm dishes and subcultured in standard 2D conditions for a total of 9-11 passages. At each passage, when cells reached 80-90% confluence, a fraction of cells was challenged in suspension by culture in poly-HEMA-coated plates for 24 hours, and viability was measured as described above. The remaining cells were maintained in 2D culture for the next passage. The number of cell generations was calculated as the number of times the population doubled over time using the formula: generations = $\log_2(x)$, where x equals the final cell number divided by the initial cell number.

Single clone viability assessment

Single-cell cloning was performed by serial dilution of OV90 cells in 96-well plates following the standard Corning protocol (https://www.corning.com/catalog/cls/documents/protocols/Single_cell_cloning_protocol.pdf). After 2 weeks of clonal expansion, individual clones were trypsinized and seeded in 6-well plates. Clones were maintained under attached conditions (2D) for 6 passages. The viability of each clone in suspension was analyzed at passages 1, 3, and 6 using Trypan blue exclusion after 24 hours in poly-HEMA-coated plates.

Doubling time measurement in 2D culture

250,000 cells were cultured in suspension in a 6 well poly-HEMA coated plate. Cell viability was measured using a Trypan blue exclusion assay every 24-48 hours for 10 days. Doubling time (DT) was calculated using the equation: $DT = \ln2/((\ln [CN1/CN2]) / (T2-T1))$. CN1 and CN2 are the cell number values measured in Time 1 and 2 (T1, T2) respectively.

Doubling time measurement in suspension culture

Cells (250,000) were cultured in suspension in 6-well poly-HEMA-coated plates. Cell viability was measured using Trypan blue exclusion every 24-48 hours for 10 days. Doubling time (DT) was calculated using the equation: $DT = \ln2/((\ln[CN1/CN2])/(T2-T1))$, where CN1 and CN2 are the cell number values measured at time points T1 and T2, respectively.

Seahorse mitochondrial stress test

Seahorse Mito Stress Test was performed following the UAB Bioanalytical Redox Biology (BARB) Core XF96 assay protocol. OV90 (20,000) and CAOV3 (25,000) cells from P0, P1, and P7 were seeded in XF96 cell culture microplates and cultured for 36 hours. The XF96 sensor cartridge was hydrated overnight in XF Calibrant at 37°C in a non-CO_2 incubator, then loaded with calibrant and equilibrated for 1 hour at 37°C. Prior to the assay, cells were washed and incubated in XF assay medium (DMEM base with 10 mM glucose, 2 mM glutamine, and 1 mM sodium pyruvate, pH 7.4) for 1 hour at 37°C in a non-CO_2 incubator. Injection ports were loaded with: Port A) oligomycin (1.5 μM final concentration), Port B) FCCP (0.675 or 0.9 μM final),

Port C) FCCP (1.65 or 1.8 μM final), and Port D) antimycin A and rotenone (0.5 μM each, final). The assay protocol consisted of 3 minutes mixing followed by 3 minutes of measurement after each port injection. Data analysis was performed using Seahorse Wave software version 2.6 and GraphPad Prism version 10.0.2. OCR values were normalized to cell number using SRB assay.

OXPHOS inhibitor treatment in suspension culture

OV90 or CAOV3 cells (250,000) from either P0 (parental) or P7 (AnR, having undergone 7 cycles of attachment-detachment) were cultured in poly-HEMA-coated 6-well plates in their respective growth media. Cells were treated with DMSO (vehicle control), 1.5 μM oligomycin (Sigma Aldrich, cat# O4876-5MG), or IM156 (MedChem Express, cat# HY-136093A) at 20 μM for CAOV3 or 15-25 μM for OV90, and incubated for 72 hours. After treatment, cells were collected and trypsinized with 10× Trypsin for 10 minutes to generate single-cell suspensions. Cell death and apoptosis were measured by flow cytometry using Annexin V/PI staining as described above.

Immune-Mediated Tumor Killing Assay

CD8+ T cells were isolated from healthy donor peripheral blood mononuclear cells (PBMCs, Precision for Medicine) using the Miltenyi CD8+ T cell isolation kit (cat# 130-096-495) according to the manufacturer's instructions. Isolated T cells were cultured at 500,000 cells per well in RPMI supplemented with 10% FBS and 30 U/ml recombinant human IL-2. For T cell activation, 1×10^6 T cells were incubated with 25 μl of Dynabeads Human T-Activator CD3/CD28 beads (Gibco, cat# 11161D) for 48 hours. Tumor cells (10,000 OV90 or CAOV3 cells from parental P0, P1, or P7 populations) were seeded in 24-well plates and allowed to adhere for 24 hours before adding activated T cells at a 10:1 effector-to-target ratio. Surviving tumor cells were counted at 24 and 48 hours (for OV90) or 24 hours (for CAOV3) using Trypan blue exclusion after gentle washing to remove non-adherent T cells. For apoptosis assessment, tumor cells were fixed and stained with cleaved caspase-3 antibody (Cell Signaling, cat# 9661T, 1:100 dilution) after T cell co-culture (12 hours for CAOV3, 48 hours for OV90). Cleaved caspase-3-positive cells were quantified using ImageJ from images captured on a Nikon TE2000 confocal microscope.

Drug Treatments

IC50 determination

Ovarian cancer cells were seeded in 96-well plates at the cell densities indicated in Table 8 and incubated until reaching 50-60% confluence. Cells were treated with 8 serial dilutions of drugs (cisplatin, doxorubicin, paclitaxel, IM156, SNX631, or THZ1) in complete growth medium for 48 hours to 7 days depending on the drug. Cell viability was assessed by SRB assay and absorbance was measured at 570 nm using a Synergy H1 microplate reader (BioTek). IC50 values were calculated using nonlinear regression (four-parameter dose-response curve) in GraphPad Prism version 10.0.2.

SNX631 and THZ1 treatment during cyclic culture

For CDK8/19 inhibitor studies during anoikis adaptation, cells were cultured in tissue culture-treated plates until reaching 80% confluence. Following trypsinization, 250,000 cells were seeded in poly-HEMA-coated 6-well plates in medium containing either DMSO (vehicle control) or the indicated concentrations of SNX631 (Senex Biotechnology, Columbia, SC) or THZ1 (MedChem Express). After 24 hours in suspension, live cell count/viability was measured using Trypan blue exclusion. Surviving cells (500,000) were seeded in 60 mm dishes in the presence of either DMSO, SNX631, or THZ1 and cultured until reaching 80% confluence. This cycle of attachment-detachment in the presence of inhibitors was continued as described in the figures.

SNX631 pretreatment and reversal studies

For reversal studies, 800,000 OV90 parental (AnS) or AnR cells were seeded in 60 mm dishes and treated with either DMSO or 500 nM SNX631 for 96 hours under standard 2D culture conditions. Following pretreatment, cells were trypsinized and 250,000 cells were transferred to poly-HEMA-coated 6-well plates in medium containing either DMSO or 500 nM SNX631 for 24 hours. Live cell count/viability was measured using Trypan blue exclusion.

Transwell fibronectin migration assay

Transwell inserts with 8 µm pore membranes (Greiner bio-one, cat# 662638) in 24-well plates were coated with 10 µg/ml fibronectin in sterile deionized water and incubated at 37°C for 2 hours. Cells were suspended in 100 µl serum-free medium and added to the upper chamber (cell numbers and incubation times are specified in Table 2). The lower chamber was filled with 600 µl of complete medium containing serum as a chemoattractant. Plates were incubated at 37°C with 5% CO2 for the indicated duration (6-24 hours depending on cell line). After incubation, medium was removed and transwells were washed twice with PBS. Non-migrated cells on the upper surface of the membrane were removed with a cotton swab. Migrated cells on the lower surface were fixed with 4% paraformaldehyde (Avantor, cat# S898-07) at room temperature for 5 minutes, permeabilized with 100% ethanol for 20 minutes, and stained with 0.5% crystal violet (Alfa Aesar, cat# B21932-22) for 15 minutes. Transwells were washed with deionized water and air-dried overnight at room temperature. Membranes were cut out and mounted on glass slides using Permount (Fisher Scientific, cat# S70104). Images were captured using an EVOS M7000 inverted microscope (Thermo Fisher) and quantified using ImageJ.

Animal Studies

Anoikis resistance xenograft studies (OV90)

Female SCID mice (8 weeks old) were injected intraperitoneally with OV90-LUC-GFP cells derived from either P0 (parental/AnS) or P7 (AnR, following 7 cycles of detachment). Cells were expanded in tissue culture-treated plates and counted immediately before injection to ensure viability. Live cells (5×10^6) in 200 µl PBS were injected i.p. Tumor growth was monitored by bioluminescence imaging (IVIS Spectrum, PerkinElmer) every 10 days until day 39-40. For imaging, mice received i.p. injection of D-Luciferin (150 µg/ml in PBS) and were imaged 10-15 minutes post-injection under isoflurane anesthesia. At the terminal endpoint, lungs were harvested, incubated in D-Luciferin solution, and imaged ex vivo for metastasis assessment. Additional terminal analyses included measurement of ascites volume (by syringe aspiration) and total tumor weight (sum of all visible tumor nodules).

Syngeneic ID8 studies

Female C57BL/6J mice (8 weeks old) received i.p. injection of ID8-EMD cells, either parental (P0) or after 7 cycles of detachment (P7). Cells were expanded in tissue culture-treated plates and 10×10^6 live cells in 200 µl PBS were injected i.p. Mice were monitored with weight and abdominal girth measurements every week for 11.5 weeks. At endpoint, mice were euthanized and analyzed for total tumor weight, omental weight, presence of mesenteric tumors, and retrievable ascites volume.

SNX631-6 efficacy studies

Female NSG mice (8 weeks old, n=24) received i.p. injection of 5×10^6 OV90-LUC-GFP cells. Beginning at the time of injection, mice were randomized to receive either control diet or SNX631-6 medicated diet (350 ppm in chow, providing an estimated daily dose of 30-50 mg/kg; prepared by Senex Biotechnology, Columbia, SC). Tumor growth was monitored by bioluminescence imaging and body weight/abdominal girth measurements every 10 days until day 41. At endpoint, mice were euthanized and analyzed for ascites volume, total tumor weight, and omental weight. Bioluminescence data were quantified as total flux (photons/second) using Living Image software.

Molecular Analyses

RNA isolation and quality assessment

Total RNA was isolated using Trizol/Chloroform extraction method. Briefly, cells were lysed in Trizol reagent, chloroform was added for phase separation, and the aqueous phase containing RNA was collected. RNA was precipitated with isopropanol, washed with 75% ethanol, and resuspended in RNase-free water. RNA quality was validated using RNA-1000 chip on an Agilent Bioanalyzer with an RIN cutoff of ≥8 for sequencing applications.

RNA-seq bioinformatics pipeline.1.0 ug of total RNA was used for the construction of sequencing libraries. The RNA-Seq analysis of OV90 P0,P1,P3,P4, P6,P7 samples (Fig. 4) was performed by Novogene, utilizing the NEBNext UltraTM RNA Library Prep Kit for Illumina on an Illumina NovaSeq 6000. All samples contained a minimum of 24 million reads with an average number of 29.2 million reads. The FASTQ files were uploaded to the UAB High-Performance Computer cluster for secondary analysis with the following custom pipeline built in the Snakemake workflow system (v5.9.1)[1]: first, quality and control of the reads were assessed using FastQC, and trimming of the bases with quality scores of less than 20 and adapter were performed with Trim_Galore! (v0.6.4). Following trimming, read quality was re-assessed with FastQC and splice-aware mapping was performed with STAR[2] (v2.6.0c, with `2-pass` mode) using the GENCODE GRCh38 primary assembly and annotation GTF (release 37). Following genome mapping, BAM index files were generated with SAMtools[3] (v1.9) and quality control of aligned files was performed with RSeQC[4] (v3.0.1). Lastly, count generation was performed with `featurecounts` (with `Rsubread`[5], v.1.32.2 and R v3.5.1) and logs of reports were summarized and visualized using MultiQC[6] (v1.6). The RNA-Seq analysis of OV90 AnS and AnR samples (+/- SNX631, 2D vs 3D, Fig. 8) was performed by University of South Carolina Functional Genomics Core Facility (RRID:SCR_026178), with libraries prepared using NEBNext poly(a) mRNA magnetic isolation kit and NEB Ultra II Directional Library Prep Kit from 500 ng RNA and sequenced on NovaSeq 6000 platform. Processed reads were aligned to genome human GRCh38.p13 primary assembly genome, utilizing STAR (v 2.7.10a) and gene counts were generated by feature counts with the Homo_sapiens.GRCh38.113.gtf annotation file. Tertiary analysis was performed in R (v 4.0.2) with the DESeq2 [7] package (v1.34.0). Briefly, pre-filtering of low abundance genes was performed to keep genes that have a mean of at least 5 counts, and normalization was performed. Following count normalization, principal component analysis (PCA) was performed, and genes were defined as differentially expressed genes (DEGs) if they passed a statistical cutoff containing an adjusted p-value <0.05 (Benjamini-Hochberg False Discovery Rate (FDR) method) and if they contained an absolute $\log_2$ fold change >=1). Additional Pathway analysis was performed using GSVA 1.42.0 (72) utilizing the Hallmarks from the Human MSigDB Collections. The gene signatures for epithelial and mesenchymal gene sets were obtained from. (73) Heatmaps were made with ComplexHeatmap version 2.10.0 (74) with the normalized enrichment score from GSVA using the Euclidean distance method.

Hallmark scatterplot graphs use normalized enrichment scores from GSEA with ClusterProfiler and were plotted using GraphPad Prism. The FASTQ files of the current study have been uploaded to NCBI's Gene Expression Omnibus under accession number GSE241546. Reference for R studio: Posit team (2023). RStudio: Integrated Development Environment for R. Posit Software, PBC, Boston, MA. URL http://www.posit.co/. UpSet plots were made using ComplexUpset R package v 1.3.3 . https://cran.r-project.org/web/packages/ComplexUpset/index.html and (70)

Whole exome sequencing

Genomic DNA was isolated using DNeasy Blood and Tissue Kit (Qiagen, cat# 69504). WES library preparation and sequencing were performed by Novogene using Agilent SureSelect Human All Exon V6 Kit. Paired-end 150-bp reads were generated on an Illumina NovaSeq 6000 with a median coverage of >159×. Demultiplexed paired FASTQ files were provided for analysis. Sequence alignment, quality control, and variant calling were performed following GATK best practices using HaplotypeCaller for germline variants and Mutect2 for somatic variant calling. All samples achieved alignment rates of 99.8-99.9%. Comparison of variants between time points was performed using MAFtools (v2.10.05). (71) Code Availability: https://github.com/page22emily/RNAseq_Anoikis

Quantitative RT-PCR

Total RNA was extracted using Trizol/Chloroform method as described above. cDNA was synthesized using iScript Reverse Transcription Supermix according to the manufacturer's protocol. Quantitative PCR was performed using iTaq Universal SYBR Green Supermix on a Bio-Rad CFX96 real-time PCR system. Expression data were normalized to housekeeping genes RPL13A, HPRT, or GAPDH using the ΔΔCt method. Primer sequences for all target genes are listed in Table 6.

Western blotting

Protein lysates were prepared in RIPA buffer supplemented with protease and phosphatase inhibitors. Protein concentration was determined by BCA assay. Equal amounts of protein were

separated by SDS-PAGE and transferred to PVDF membranes. Membranes were blocked in 5% non-fat milk or BSA in TBS-T and incubated with primary antibodies overnight at 4°C: c-MYC (Cell Signaling, cat# 13987), TAP1 (Cell Signaling, cat# 49671S), MHC-I (R&D Systems, cat# NBP3-16696), pSTAT1 (Cell Signaling, cat# 9177S), STAT1 (Cell Signaling, cat# 14994), cleaved caspase-3 (Cell Signaling, cat# 9661T), and γH2AX (Cell Signaling, cat# 9718T). β-actin was used as loading control. After washing, membranes were incubated with HRP-conjugated secondary antibodies for 1 hour at room temperature. Bands were visualized using enhanced chemiluminescence and quantified using ImageJ.

Data and Code Availability

RNA sequencing data generated in this study are publicly available at NCBI Gene Expression Omnibus under accession numbers GSE241546 and GSE309005. All code for data analysis is available at https://github.com/page22emily/RNAseq_Anoikis. Supporting data values for all figures are provided in the accompanying Excel file.

Software

The following software was used for data analysis and visualization: ImageJ (NIH, RRID:SCR_003070) for image analysis, GraphPad Prism 9/10 (RRID:SCR_002798) for statistical analysis and graphing, FlowJo 10.8.1 (RRID:SCR_008520) for flow cytometry analysis, Adobe Illustrator (RRID:SCR_010279) for figure preparation, R/RStudio (Posit Software, PBC, Boston, MA) for bioinformatics analyses, Seahorse Wave 2.6 for metabolic data analysis.

TABLES

Table 1. Cell lines

CELL LINE	SOURCE	IDENTIFIER
OV90	ATCC	CRL-11732
CAOV3	ATCC	HTB-75
OVCAR3	NIH	NCI60 (0507709)
OVCAR4	NIH	NCI60
OVCAR5	NIH	NCI60
OVCAR10	Susan Murphy (Duke University, Durham, NC)	
SK-OV3	ATCC	bnHTB-77
OVCA420	Susan Murphy	N/A
HEY	Susan Murphy	N/A
HEYA8	Susan Murphy	
TYK-nu	Susan Murphy	
P76	Amir Jazaeri	N/A
P151	Amir Jazaeri	N/A
P201	Amir Jazaeri	N/A
P210	Amir Jazaeri	N/A
FT282	ATCC (Ronny Drapkin)	
IOSE141	Canadian Tissue bank	
ID8-EMD	EMD Millipore	SCC145

HEK293	ATCC	CRL-1573
EOC15	Penn state/UAB	N/A

Table 2: Commercial Kits

Reagents	Source	Catalog number
LIVE/DEAD™ Viability/Cytotoxic kit	Fisher Scientific	L3224
LookOut® Mycoplasma PCR Detection Kit	Millipore sigma	MP0035-1KT
Annexin V Apoptosis Detection Kits	eBioscience™	88-8005-74
CD8+ T cells isolation kit	Miltenyi Biotec	130-096-495
DNeasy Blood and Tissue Kit	Qiagen	69504

Table 3: Antibodies

Antibodies	Source	Catalog number
Ki-67 (8D5) Mouse mAb	Cell signaling	9449
Goat anti-Mouse IgG1 Cross-Adsorbed	Invitrogen	A-21125

Secondary antibody Alexa Fluor™		
Cleaved caspase 3	Cell signaling technology	9661T
YH2AX	Cell signaling technology	9718T
annexin V	Bio Legend	640906
PI	Invitrogen	00-6990-42
pSTAT1	Cell signaling technology	9177S
STAT1	Cell signaling technology	14994
MHC1	R&D	NBP3-16696
TAP1	Cell signaling technology	49671S
MYC	Cell signaling technology	13987

Table 4: Other reagents and recombinant constructs

Reagent	Source	Catalog number
ProLong™ Gold Antifade Mountant	Invitrogen	P36930

Dynabeads® Human T-Activator CD3/CD28	Gibco	11161D
Crystal violet	Alfa Aesar	B21932-22
Permount	Fisher scientific	S70104)
Oligomycin	Sigma Aldrich	O4876-5MG
IM156	MedChem Express	HY-136093A
THZ1	MedChem Express	HY-80013
SNX631	Senex Biotechnology	
SNX631-6 (Diet)	Senex Biotechnology	
Poly-HEMA	Sigma Aldrich	P3932-25G
hTERT	Gen Target	LVP1130-Puro-PBS
SV40 large T-antigen lentivirus	Gen Target	LVP016-Hygro
siC-MYC	Thermo fisher	4392420 (s9130)
siC-MYC	Thermo fisher	4392420 (s9129)
siNTC	Thermo fisher	4390843

Table 5: Software used

Software	Source	Identifier
ImageJ	NIH	ImageJ,<br>RRID:SCR_003070
Prism9	GraphPad	GraphPad Prism<br>(RRID:SCR_002798)(
Adobe Illustrator	Adobe	Adobe Illustrator,<br>RRID:SCR_010279
FlowJo		FlowJo (RRID:SCR_008520)

Table 6. qRT-PCR Primers (listed 5' to 3')

1	RPL13A F human: GGC CCA GCA GTA CCT GTT TA<br>RPL13A R human: AGA TGG CGG AGG TGC AG
2	HLA-A F human: AGA TAC ACC TGC CAT GTG CAG C<br>HLA-A R human: GAT CAC AGC TCC AAG GAG AAC C
3	HLA-B F human: CTG CTG TGA TGT GTA GGA GGA AG<br>HLA-B R human: GCT GTG AGA GAC ACA TCA GAG C
4	HLA-C F human: GGA GAC ACA GAA GTA CAA GCG C<br>HLA-C R human: ACA TCC TCT GGA GGG TGT GAG A

Table 7: Cell density for transwell fibronectin migration assay

Cell line	Initial cell density	Incubation time (hours)
OV90	100000	24
CAOV3	100000	24
OVCA420	75000	24
EOC15	100000	24
P151	75000	6

Table 8: Cell density for IC50 experiments

Cell line	Initial cell density
OV90	5000
OVCA420	2000
CAOV3	5000
EOC15	5000
