# Supplementary material for: Anoikis resistance and metastasis of ovarian cancer can be overcome by CDK8/19 mediator kinase inhibition
Source: JCI Insight. 2026 Jan 15;11(4):e192113. doi: 10.1172/jci.insight.192113 (PMC12956021; doi:10.1172/jci.insight.192113)
Supplement: Unedited blot and gel images [file jciinsight-11-192113-s205.pdf]

Figure 1G OV90

Cleaved caspase 3

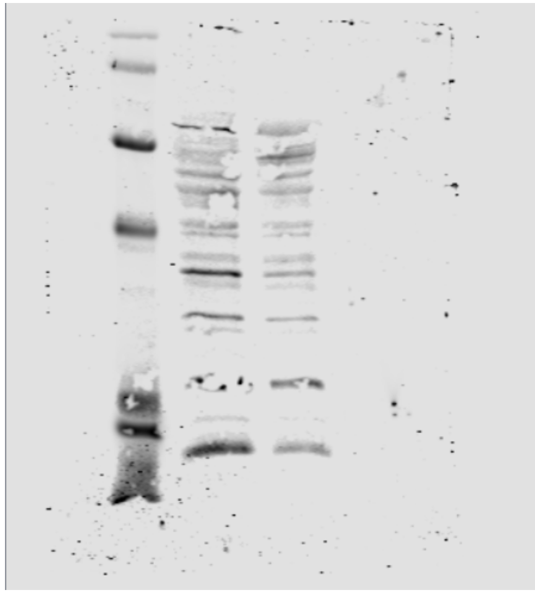

βactin

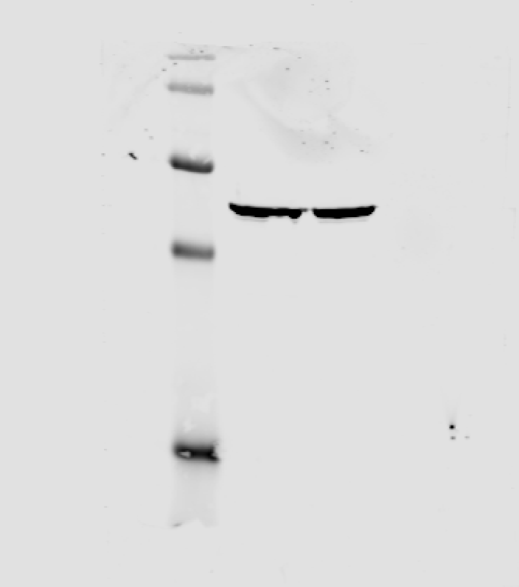

Figure 1G CAOV3

Cleaved caspase 3

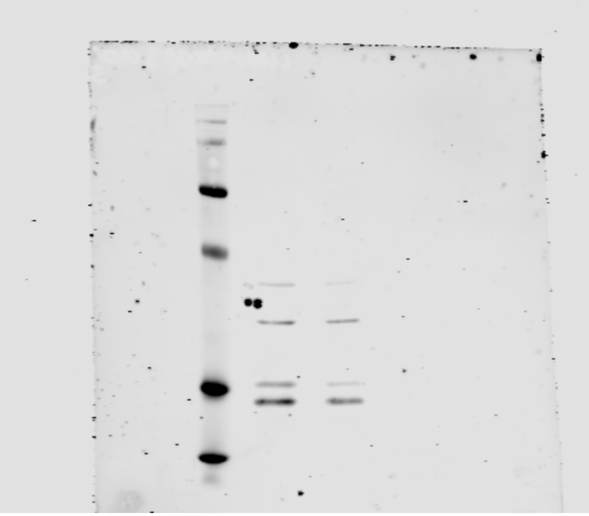

βactin

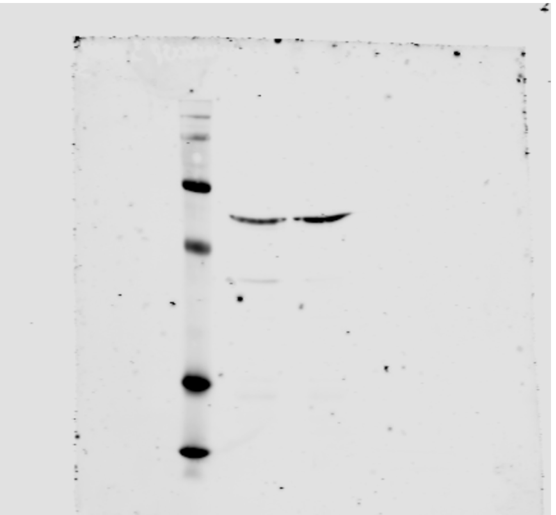

Figure 4F

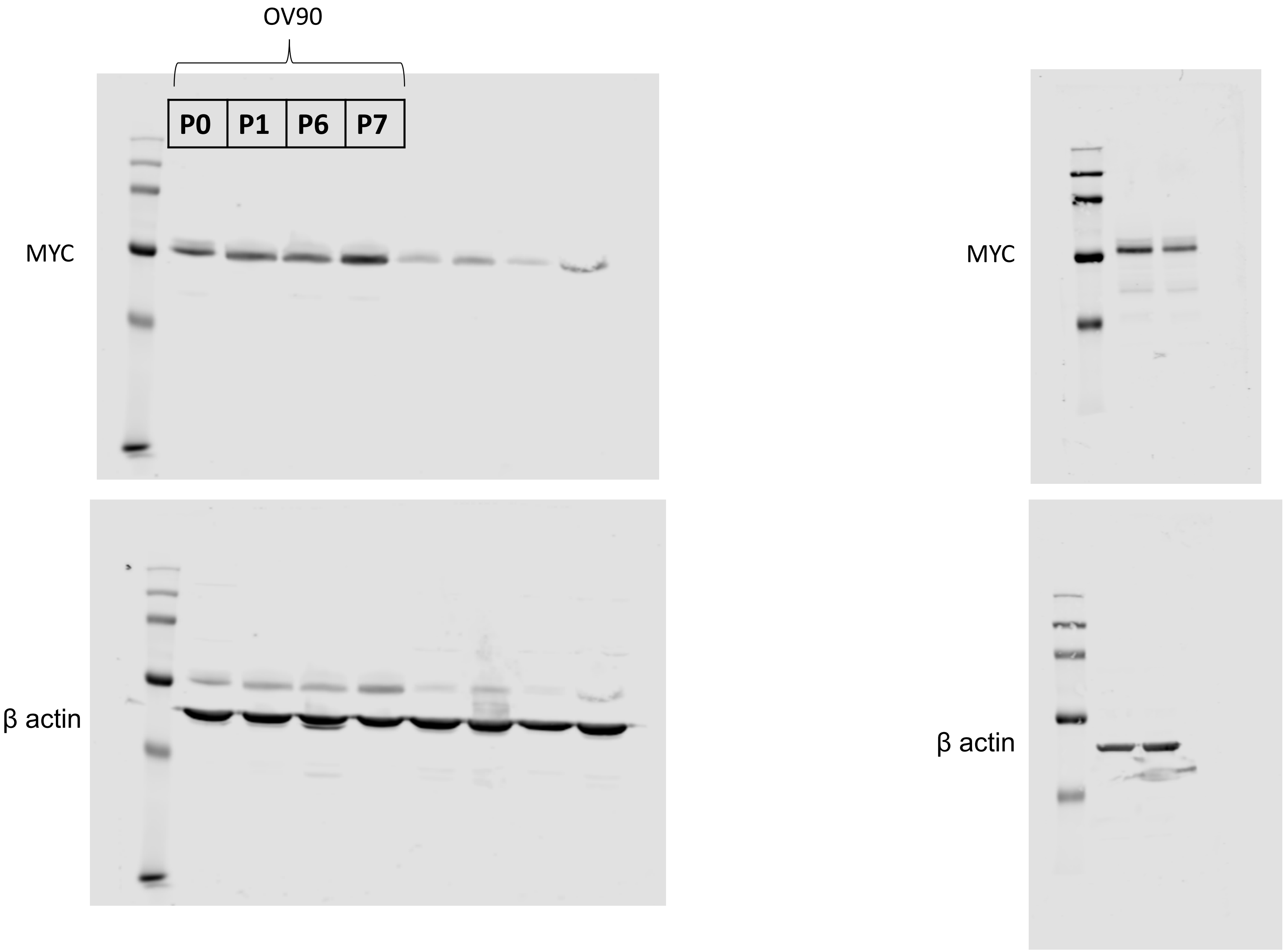

Figure 6C

OV90

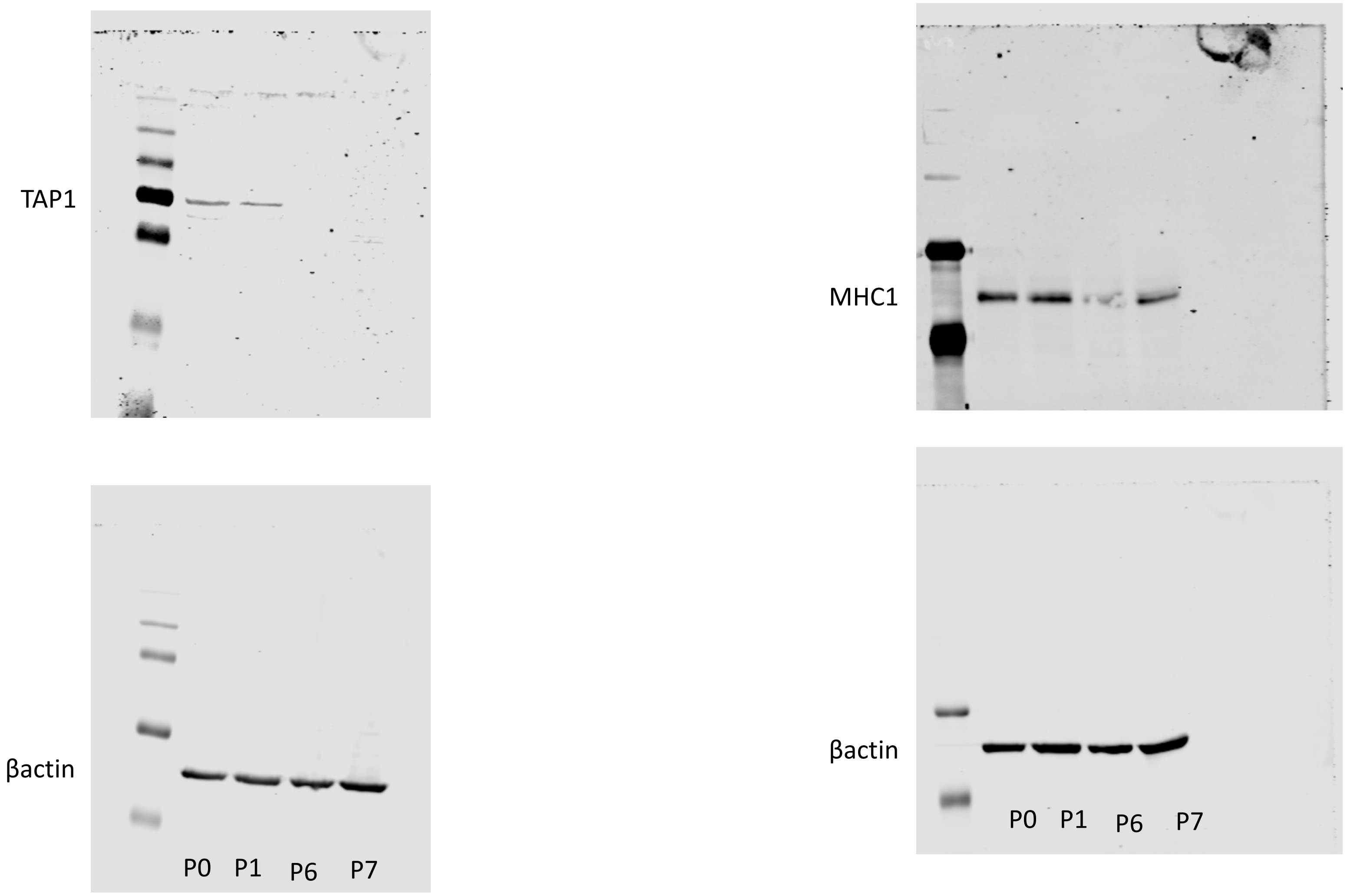

Figure 7A

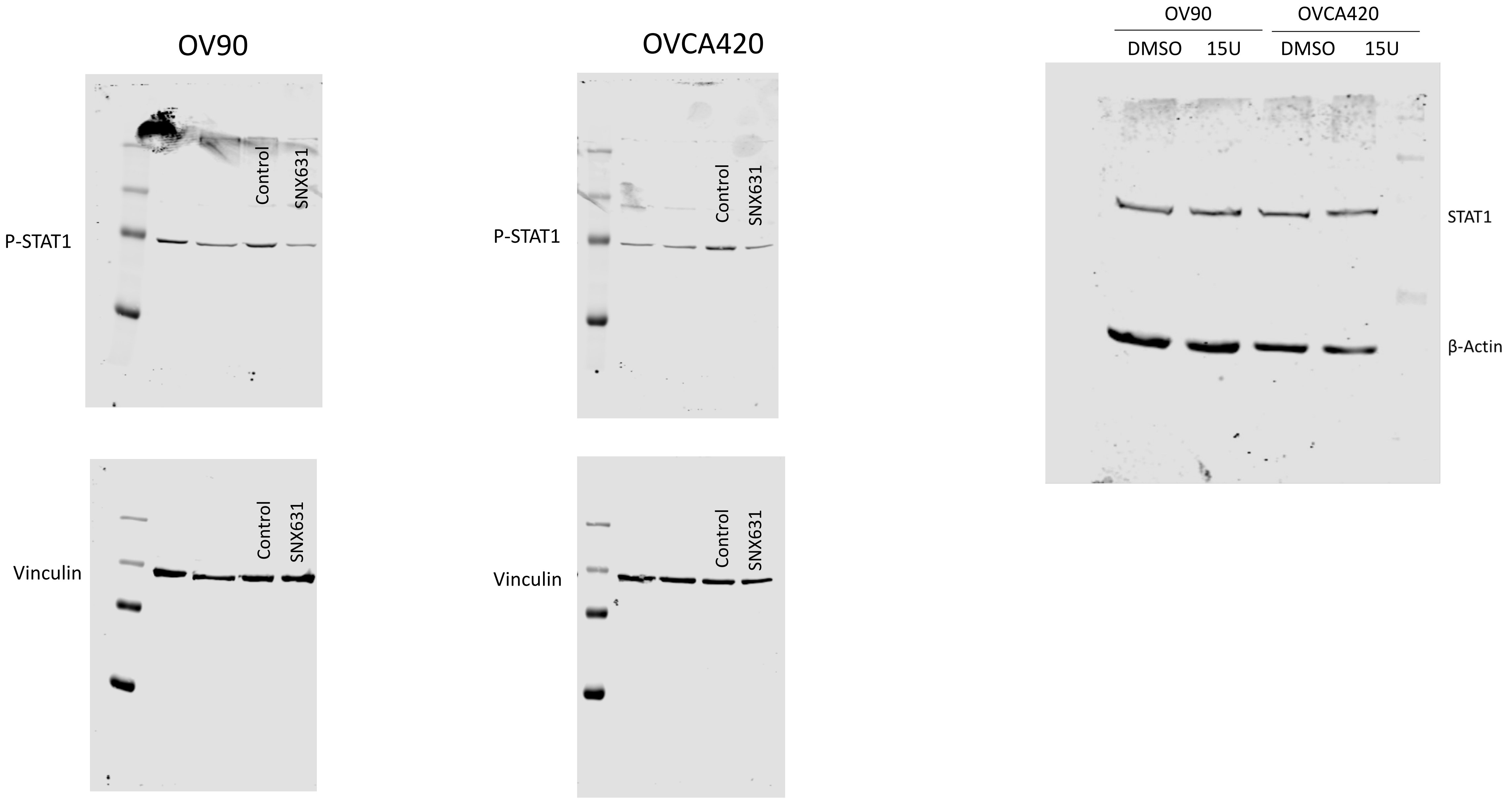
OV90
P-STAT1
Control
SNX631
Vinculin
Control
SNX631
OVCA420
P-STAT1
Control
SNX631
Vinculin
Control
SNX631
OV90
OVCA420
DMSO
15U
DMSO
15U
STAT1
β-Actin
